# Beta_2_-Adrenergic Suppression of Neuroinflammation in Treatment of Parkinsonism, with Relevance for Neurodegenerative and Neoplastic Disorders

**DOI:** 10.3390/biomedicines12081720

**Published:** 2024-08-01

**Authors:** Mario A. Inchiosa

**Affiliations:** Department of Pharmacology, New York Medical College, Valhalla, NY 10595, USA; mario_inchiosa@nymc.edu

**Keywords:** Parkinson’s disease, α-synuclein protein, Lewy bodies, epinephrine, beta2-adrenergic agonist, levalbuterol, dopaminergic neurons, neuroinflammation, eotaxin-1, VEGFa

## Abstract

There is a preliminary record suggesting that β_2_-adrenergic agonists may have therapeutic value in Parkinson’s disease; recent studies have proposed a possible role of these agents in suppressing the formation of α-synuclein protein, a component of Lewy bodies. The present study focuses on the importance of the prototypical β_2_-adrenergic agonist epinephrine in relation to the incidence of Parkinson’s disease in humans, and its further investigation via synthetic selective β_2_-receptor agonists, such as levalbuterol. Levalbuterol exerts significant anti-inflammatory activity, a property that may suppress cytokine-mediated degeneration of dopaminergic neurons and progression of Parkinsonism. In a completely novel finding, epinephrine and certain other adrenergic agents modeled in the Harvard/MIT Broad Institute genomic database, CLUE, demonstrated strong associations with the gene-expression signatures of anti-inflammatory glucocorticoids. This prompted in vivo confirmation in mice engrafted with human peripheral blood mononuclear cells (PBMCs). Upon toxic activation with mononuclear antibodies, levalbuterol inhibited (1) the release of the eosinophil attractant chemokine eotaxin-1, which is implicated in CNS and peripheral inflammatory disorders, (2) elaboration of the tumor-promoting angiogenic factor VEGFa, and (3) release of the pro-inflammatory cytokine IL-13 from activated PBMCs. These observations suggest possible translation to Parkinson’s disease, other neurodegenerative syndromes, and malignancies, via several mechanisms.

## 1. Introduction

The principal objective of this study evolved from several early observations that related agonist activity of certain synthetic drugs to selective beta2-adrenergic activity, showing beneficial improvement in patients with Parkinson’s disease (PD). These were followed at a considerably later time by more extensive studies showing a link between beta-2 adrenergic activity and suppression of transcription of the α-synuclein gene (*SNCA*), which is implicated in the formation of Lewy bodies that characterize PD progression. As reviewed here, several epidemiological studies have shown a decreased association between beta-2 adrenergic treatments and the prevalence of PD in human populations.

Also, lifestyle characteristics such as purposeful exercise, nicotine intake from tobacco use, and consumption of caffeine-containing beverages have been extensively shown to decrease the incidence of PD in a quantitative correlation relative to the extent of exposure to these interventions. Evidence has been presented for a relation between the plasma concentrations of the prominent b2-adrenergic neurotransmitter epinephrine in relation to these lifestyle practices and an association with PD incidence or progression. The investigations at this point turned directly into the genomic modeling of epinephrine in the Broad Institute Harvard/MIT database, CLUE, to examine its similarity (“connectivity”) with gene-expression characteristics of its large collection of chemicals, drugs, and genetic variables. This evaluation revealed the novel unexpected finding of a prominent similarity between the gene-expression output of epinephrine and natural and synthetic glucocorticoids. In view of the anti-inflammatory properties of glucocorticoids, this prompted biological studies of the anti-inflammatory activity of synthetic beta2-adreneric and related adrenergic agents in human peripheral blood mononuclear cell culture-engrafted (PBMC) mice. These studies and their implications suggested the possible clinical translation of the findings.

### 1.1. Early Observations

Magistrelli and Comi [1] reviewed the early history of reports of an association between treatment with β_2_-adrenergic receptor (β2AR) agonists and a therapeutic benefit in patients with Parkinson’s disease (PD). Three open-label studies were noted, dating from 1992, 1994, and 2003, with the use of salbutamol as an adjunct to levodopa in a total of 25 patients. In the earliest study (nine PD patients), there were significant increases in “on time” and shortening of response latency [2]. The 1994 study with eight PD patients [3] showed statistically significant improvement in PD tests for manual dexterity and time to rise and walk 20 feet and return and sit again; the 2003 study with eight PD patients also found improvement in manual dexterity and in Unified Parkinson’s Disease Rating Scale (UPDRS) scores [4].

### 1.2. α-Synuclein Gene (SNCA) and PD

The observations made in these earlier studies have advanced considerably with the recognition that β2AR ligands modulate the transcription of the α-synuclein gene (*SNCA*) and that the gene product α-synuclein (α-syn) is implicated in the development of PD [1,5]. PD pathology is clinically correlated with the extent of excessive expression of *SNCA* and synthesis of α-syn, which accumulates intracellularly in Lewy bodies in the brains of PD patients.

In addition to PD, synucleinopathies include the neurodegenerative diseases Lewy bodies dementia and multiple system atrophy. All three demonstrate cellular accumulation of α-syn and loss of dopaminergic neurons [6].

Mittal et al. [5] screened 1126 FDA-approved drugs and a variety of health supplements in cultured human neuroblastoma cells (SK-N-MC) for their ability to reduce transcription of *SNCA* mRNA in a four-stage gene expression assay. The highest efficacies were found in three β2AR agonists, metaproterenol, clenbuterol, and salbutamol, and, interestingly, in the drug riluzole which has been approved by the FDA for intervention in amyotrophic lateral sclerosis, where it was found to decrease dopaminergic degeneration in a PD rat model. All three β2AR agonists caused statistically significant relative reductions in *SNCA* mRNA abundance and α-syn protein abundance in SK-N-MC cells [5]. Of particular relevance to PD pathology and loss of dopaminergic neurons, the investigators also found that intraperitoneal clenbuterol treatment for 24 h reduced expression of *SNCA* in the substantia nigra of C57BL/6J wild-type mice, with decreases in nigral mRNA and α-syn protein. Additionally, higher cellular levels of α-syn produced mitochondrial dysfunction and production of superoxide anions and other reactive oxygen species (ROS); clenbuterol treatment in neuronal precursor cells (iPSC-derived) from a PD patient suppressed these changes [5].

### 1.3. PD Causation from Neuroinflammation

A number of studies have focused on the importance of inflammatory processes in neurodegenerative diseases, including PD. In connection with this, there is considerable interest in the apparent effect of β2AR signaling on immune cells in suppressing the inflammatory potential of microglia and macrophages, and in T cells and B cells. Signaling through this receptor can influence the inflammatory responses of these cells [1,5,7,8,9,10,11,12,13].

The role of neuroinflammation in the initiation and progression of PD is a major focus of the present investigations. The functional role of β2AR in biological responses has been further elucidated by the development of “short-acting” and “long-acting” relatively selective β2AR agonists that have little β1AR efficacy. These drugs have been used primarily for the treatment of bronchial asthma and chronic obstructive pulmonary disease (COPD). The nomenclature for these agents in the literature includes either the “United States Adopted Name” (USAN) or the “International Nonproprietary Name” (INN), usually depending upon the country of origin of the research or the date of the publication. The designated names of the principal drugs that we investigated were as follows: Albuterol sulfate is the USAN for a short-acting bronchodilator that has the INN salbutamol sulfate. Albuterol is a racemic mixture of equal quantities of S and R isomers; all of the β2-adrenergic activity resides in the R isomer. Some proprietary names for albuterol sulfate are Ventolin, Proventil, and ProAir. The pure R-isomer has the USAN of levalbuterol HCl (a proprietary formulation as levalbuterol tartrate is named Xopenex); its INN is levosalbutamol (it is also referred to as R-salbutamol). Arformoterol is the USAN for the pure R-isomer of a long-acting β2AR agonist; the racemic mixture of the drug is named formoterol. The bronchodilator drug theophylline was also studied; it does not have β2AR agonist activity but inhibits the metabolism of cyclic AMP (cAMP) by phosphodiesterase enzymes, thereby preserving intracellular levels; cAMP is the initial signaling messenger that results from activation of both β1- and β2-ARs.

It is important to note the differences in the expected anti-inflammatory activity of β2AR agonists regarding whether they are racemic mixtures, such as albuterol, or the pure R-isomer, such as levalbuterol. In addition to the fact that the R-isomer has all the bronchodilator activity of albuterol, it also has all the anti-inflammatory activity. The S-isomer of albuterol has pro-inflammatory activity and partially, completely, or overwhelmingly negates the anti-inflammatory activity of the R-isomer. Mazzoni et al. [14] demonstrated a hyperresponsiveness to histamine in guinea pig airways following pretreatment with S-albuterol. This is apparently related to the capacity of that isomer to increase smooth muscle proliferation, as observed in human bronchial cell cultures [15]; levalbuterol decreased cell proliferation compared with control cultures enriched with 5% fetal bovine serum. Baramki et al. [16], Chorley et al. [17], and Wang et al. [7] have all confirmed the anti-inflammatory effects of levalbuterol (R-albuterol) and the pro-inflammatory and compromising effects of S-albuterol on those of levalbuterol.

### 1.4. Epidemiological Associations between Beta2-Adrenergic Effects and PD

Several large epidemiological studies revealed findings that the clinical use of β2AR agonists (primarily for patients with asthma) was associated with a decreased incidence of PD compared to the general population and that the use of a β2AR antagonist (primarily for the treatment of hypertension) led to an increased incidence. Mittal et al. [5] studied the Norwegian Prescription Database over an 11-year period, for the entire population alive on 1 January 2004 (n = 4.6 million). Patients treated with salbutamol (albuterol) showed a decreased PD risk; the rate ratio (incidence with salbutamol/incidence without exposure to salbutamol) was 0.66 (95% confidence interval, CI of 0.58 to 0.76). Patients treated with propranolol, a β1-, β2AR antagonist, had a markedly increased rate ratio of PD, up to 2.20 (95% CI of 1.62 to 3.00).

Using health records from the major Israeli health provider, Gronich et al. [18] studied medical records of 1,762,164 individuals who did not have a PD diagnosis on 1 January 2004 and followed their health histories until 30 June 2017. Each participant who was treated with salbutamol was compared with 10 cohorts as controls and showed a decreased rate ratio of 0.89 for the development of PD (0.82–0.96); *p* = 0.004. Participants treated with propranolol showed an increased PD rate ratio of 2.60 (2.40–2.81); *p* < 0.001. A special feature of this study [18] was that it included a number of participants that were treated with a group of β1-selective antagonists instead of the β1-, β2AR antagonist propranolol. A total of 3032 participants received either metoprolol, atenolol, or bisoprolol. Since these drugs leave β2ARs largely available, it would be expected that they would be chronically stimulated by endogenous epinephrine released during daily activities and exercise. These participants showed no change in PD rate ratio compared with their cohort controls; 1.00 (0.95–1.05); *p* = 0.94. All the above epidemiological studies consistently demonstrated that drugs that stimulated the β2AR receptor, or interventions that left it available for activation, decreased the incidence of PD.

Chen et al. [19] conducted a meta-analysis on the association between β-adrenoceptor drugs and Parkinson’s disease and distilled 640 studies down to 8 that were appropriate for meta-analysis. Salbutamol yielded a decreased rate ratio of 0.888 for PD (0.822–0.960); *p* = 0.003. When the studies that used one or more of the β2AR agonists were analyzed together, the rate ratio for PD was still lowered: 0.840 (0.714–0.987); *p* = 0.035. This included the β2-selective agonists salbutamol, formoterol, salmeterol, and terbutaline. It must be noted that all these drugs were the racemic mixtures; in the studies noted above, only the levo (R-)isomers were shown to have anti-inflammatory activity, while the S-isomer portion was actually proinflammatory and compromised the effects of the R-isomer. Thus, the decreased incidence of PD in participants that received β2AR agonists may have been greater if the single R-isomer agents had been studied.

### 1.5. Life-Style Factors That May Influence the Incidence or Progression of PD

There is considerable evidence that certain lifestyle practices delay the initiation of PD and the rate of its progression. These include physical exercise, nicotine exposure from smoking, and intake of caffeine from consumption of coffee or tea. In a potential connection with the present study, these lifestyle interventions are associated with an increased outflow of the sympathetic nervous system and elevations in circulating levels of epinephrine, with its prominent β2AR-agonist activity.

Exercise programs are a major therapeutic recommendation on the websites of the Parkinson’s Foundation and the Michael J. Fox Foundation to forestall the progression of disease and to ameliorate the symptoms of PD. Moore et al. [20] reviewed the extensive literature showing the beneficial effects of physical exercise in human and animal models of PD. They also presented the projected design of a Phase II trial, “Study in Parkinson Disease of Exercise”, (SPARX), which was intended to serve as a pilot study to determine whether a larger study on graded intensity of exercise was warranted. It included patients who were at an early stage of PD and were not on dopaminergic therapy or, if so, for only a short period. There were three groups: high-intensity treadmill exercise for 30 min, four times/week at 80–85% of maximum heart rate; moderate-intensity exercise for 30 min, four times/week at 60–65% of maximum heart rate; and usual care for a waiting-list group who were eligible for later inclusion in the study. The ability to identify treatment effects was complicated by the fact that the usual care group were permitted to continue their current exercise programs since, as noted above, the value of exercise in PD was already appreciated and it would not have been ethical to prevent the control group from gaining the possible benefits of their existing programs. The groups were to be maintained and monitored for a period of 6 months.

Schenkman et al. [21] reported the results of a Phase II pilot trial that included a total of 128 participants. There were improvements in the Movement Disorder Society revised Unified Parkinson Disease Rating Scale (MDS-UPDRS) in both treadmill groups, but statistical significance was reached only at the high-intensity level. Recruitment is now ongoing for the SPARX3 trial that will be conducted at 29 sites in the United States and Canada (Study No. NCT04284436; sparx3pd.com; accessed 26 July 2024) with 370 participants. This study will have considerably more statistical power since there will only be two groups divided into the two intensity levels; the changes in MDS-UPDRS scores will be evaluated for each participant, comparing their initial values and those at the end of the study period. The SPARX3 trial has been delayed by the COVID-19 pandemic and completion of recruitment is now estimated to be in July 2025, with the results available approximately 18 months after close of the study.

The studies by Kjaer et al. [22] on epinephrine release with exercise are of relevance in relation to the evolving focus on the intensity of exercise as therapy in PD. Their findings in a series of studies showed a direct relationship between exercise intensity in healthy adults and epinephrine concentrations in arterial plasma. Four levels of exercise intensity were studied, based on the individual subjects’ maximal oxygen uptake (VO_2_MAX). Resting epinephrine concentration averaged 0.5 nmol/L. Plasma epinephrine concentration after 60 min at 40–56% VO_2_MAX averaged 1.6 nmol/L; after 25 min at 60–70% VO_2_MAX, 2.2 nmol/L; after 25 min at 82–89% VO_2_MAX, 4.5 nmol/L; and after 4 min at 100–110% VO_2_MAX, 8.7 nmol/L.

Tobacco smoking has a long and established negative association with the onset and progression of PD [23,24,25,26,27]. Meta-analysis by Hernan et al. [27] that included 44 case-controlled studies and four cohort studies showed a relative risk of PD between current smokers and individuals that had never smoked of 0.39 (95% confidence interval of 0.32–0.47). The pharmacological effects of nicotine inhalation on the sympathetic nervous system would appear to be a convincing explanation for this protective effect. In addition to stimulating outflow of the sympathetic nervous system from the brain, nicotine peripherally activates nicotinic receptors in sympathetic ganglia to release norepinephrine from sympathetic nerve endings and also generates release of epinephrine by stimulation of nicotine receptors in the adrenal medulla.

Cryer et al. [28] studied the effects on catecholamine plasma concentrations in 10 male subjects in conjunction with two trials of smoking 5.5 cm of a cigarette in a 10 min period. The men were chronic smokers but had fasted overnight and had not smoked until the time of the experiments. Plasma concentrations of norepinephrine and epinephrine rose rapidly during the smoking period to maxima at about the 10 min time point. The average epinephrine concentration of 44 ± 4 (mean; S.E.) pg/mL before smoking rose to 113 ± 27 pg/mL (*p* < 0.05) at 10 min; it remained noticeably above the basal level for the next 20 min until measurements were halted. Grassi et al. [29,30] also studied the acute effects of cigarette smoking on epinephrine plasma levels. Their protocol required 48 h of abstinence from smoking and completion of the cigarette in a 5 min period. Although the smoking period was half that in the previous study [28], epinephrine concentrations increased from 25 ± 8.6 (mean; S.E.) pg/mL before smoking to 47 ± 11.5 pg/mL (*p* < 0.01) at the end of the period.

The relationship of caffeine consumption to a decreased incidence of PD has a long and consistent documentation [27,31,32,33,34,35,36]. Tan et al. [32], in a completely Chinese population, found that 10 years of coffee consumption at three cups/day was associated with a decreased odds ratio of 0.78 for PD (95% CI 0.66–0.93) *p* = 0.006, and for 10 years of tea drinking at the cups/day, the odds ratio was 0.72 (95% CI 0.56–0.94) *p* = 0.014). Ren and Chen [36] reviewed several large prospective studies and meta-analyses that showed strong evidence of the negative association between caffeine consumption and PD. Notably, a meta-analysis by Qi and Li [35] that involved a total of 901,764 participants from 13 studies showed a maximum benefit in PD incidence reduction with approximately three cups of coffee/day, at a relative risk of 0.72 (95% CI 0.65–0.81). The same review [36] presented evidence from animal studies that caffeine may reduce neuroinflammation and its attendant loss of dopaminergic neurons. Also, in α-syn animal models of PD, caffeine administration was associated with a decreased aggregation of α-syn, the component of Lewy bodies.

Finally, consistent with the above observations for physical exercise and cigarette smoking, caffeine consumption results in increases in plasma epinephrine. In a precisely controlled clinical study, Robertson et al. [37] demonstrated that 250 mg of caffeine in non-coffee drinkers resulted in a significant increase in plasma epinephrine concentration one hour after consumption, from 36 ± 5 pg/mL (mean, S.E.) for a control beverage to 89 ± pg/mL with caffeine; *p* < 0.001. Plasma epinephrine concentrations were still statistically different from controls in the final measurements 3 h after caffeine consumption.

Benowitz et al. [38] observed similar findings for caffeine consumption of 2 and 4 mg/kg, in a cross-over study design in coffee drinkers who had abstained for 3 days before the study. The results expressed as maximum changes in epinephrine concentrations were 35 ± 19 (mean, S.E.) pg/mL for the 2 mg/kg dose and 63 ± 18 pg/mL for 4 mg/kg. Both doses were statistically different from controls (*p* < 0.05) and the 4 mg/kg dose was statistically greater than the 2 mg/kg dose (*p* < 0.05). Measurements were made over a 180 min period and the areas under the time × plasma concentration curves (AUCs) were also statistically and markedly different from the controls, with the 4 mg/kg dose again statistically different from the 2 mg/kg dose (differences of *p* < 0.05).

### 1.6. Genomic Modeling of the Potential Anti-Inflammatory Effects of Epinephrine

The evidence outlined above on the possible role of β2AR-agonist activity on (1) reducing the expression of *SNCA* mRNA and its protein product α syn, (2) the reduction in neuroinflammation and loss of dopaminergic neurons, (3) epidemiological associations with decreased incidence of PD in their routine clinical use, and (4) lifestyle factors such as physical exercise, cigarette smoking, and caffeine consumption that are negatively associated with PD incidence, and which all result in increased plasma epinephrine concentration, prompted an attempt to explore a possible link between the gene-expression characteristics of epinephrine and anti-inflammatory potential.

Epinephrine was modeled in the Harvard/MIT Broad Institute genomic database, CLUE [39]. It was found to have a remarkably coherent similarity in gene-expression activity to the human glucocorticoid cortisol (hydrocortisone), which has profound anti-inflammatory activity. Of particular interest, this property was not shared with norepinephrine, which is known to have minimal β2AR-agonist activity. These observations led to the investigation of the potential anti-inflammatory effects of a series of β2AR agonists in Jackson Laboratory human peripheral blood mononuclear cell (PBMC)-engrafted mice.

### 1.7. Anti-Inflammatory Studies in the Cytokine Release Syndrome (CRS) Assay

A CRS assay in humanized mice from Jackson Laboratories was utilized to evaluate the anti-inflammatory potential of several of the β2AR agonists and related compounds noted above, primarily to evaluate their capacity to protect against and modulate neuroinflammation. This assay employs immunodeficient mice that have been engrafted with human peripheral blood mononuclear cell cultures (PBMCs) that are then challenged with several monoclonal antibodies (mAbs) that are known to potently induce clinically injurious cytokine release [40,41]. The mice can be pre- and chronically treated with agents of interest for anti-inflammatory effects in conjunction with the mAb challenges. The Jackson Laboratories, Bar Harbor, Maine website provides additional details of the CRS assay.

The direct β2AR agonists albuterol, levalbuterol, and arformoterol and the indirect agonist theophylline, all noted above to increase cellular levels of cAMP, were studied in the mouse CRS assays. Phenoxybenzamine, a noncompetitive antagonist of alpha2-adrenergic agonist activity that results in increased cellular levels of cAMP [42], was also assayed. All the compounds except albuterol, the racemic mixture of S and R albuterol, showed anti-inflammatory potential in the CRS assay.

The possible anti-inflammatory activity of beta2-adrenergic agonists, as suggested by their gene-expression similarity to that of glucocorticoid receptor agonists, became a principal aim of these investigations. The hypothesis was tentatively confirmed by the inhibitory effects of beta2-adrenergic drugs on the release of several cytokines and chemokines in activated human PBMC-engrafted mice.

## 2. Materials and Methods

### 2.1. Analyses of Gene-Expression Signatures of β2-Adrenergic Agonists and Related Compounds in the Harvard/MIT Broad Institute Genomic Database, CLUE

Both the earlier online Broad Institute CMap genomic dataset and the new cloud-based CLUE platform are based on the gene-expression signatures that result from the perturbation of actively proliferating cells that are malignant. The earlier online platform showed predictions of antitumor and histone deacetylase activity of phenoxybenzamine [43]. The strength of these predictions was extended when phenoxybenzamine was profiled on the cloud-based platform [44]. Both references provide additional details of the methodology.

The database and associated software are accessible at https://clue.io (accessed on 1 April 2024). The drugs, chemical agents, and genetic inputs are referred to as “perturbagens” [39,45]. The present analyses focused on gene-expression signatures located in the Touchstone dataset (accessed in the “Tools” menu), which was selected because it represents a set of thoroughly annotated small-molecule perturbagens that would be expected to be relevant to comparisons with the possible effects of β2-adrenergic agonists; see: https://clue.io/connectopedia/tag/TOUCHSTONE (accessed on 29 July 2024). The Touchstone dataset contains a total of approximately 8400 perturbagens that produced gene signatures that were generated from testing on a panel of 9 proliferating malignant cell lines; those cell lines are detailed in Table 1.

In almost all instances, the perturbagens were tested at a concentration of 10 µM, allowing comparison of the strength of the connectivity of gene expression between agents at equimolar concentrations.

It should be noted that the dataset in the earlier online platform of CMap was based almost entirely on perturbations in only 3 tumor cell lines, MCF7 (human breast adenocarcinoma), PC3 (human prostate adenocarcinoma), and HL60 (human promyeloblast); the first 2 cell lines are among the 9 in the current CLUE platform and consistency among the gene-expression signatures for the different cell lines is strengthened by the inclusion of additional cell lines.

The scoring value obtained in the gene expression analyses is termed “tau”; it ranges from 100 to −100 and is a measure of the connectivity between the gene-expression signature of the perturbagen of interest and those of the other 8400 perturbagens in the database. A positive tau indicates a relative similarity between two perturbagens or groups of perturbagens, while a negative score indicates relative opposing gene signatures. Thus, for example, a tau score of 95 indicates that only 5% of the signatures in the database have connectivity higher than that of the perturbagen being profiled; see https://clue.io/connectopedia/connectivity_scores (accessed on 29 July 2024). Positive scores above 90 are generally considered worthy of consideration as representing possibly important similarities in gene expression. The gene-expression data outputs from the analyses place emphasis on this range.

The Touchstone database returns two data formats in response to a query about the similarities or differences between a perturbagen of interest and the other perturbagens in the database. The compound being searched against all other entries in the Touchstone database is termed the “index”. A “heatmap” format presents the connectivity score between the queried perturbagen and the reference perturbagens in the database for each of the 9 cell lines that have been studied. (From 7 to 9 cell lines may be chosen for a particular index analysis). The second format is the “detailed list” output. In our experience, this output provided the most convenient access to information that related to the primary focus of the present studies. The detailed list format also includes the protein targets of the individual perturbagens. Both formats provide comparison of the gene signatures of the index compound with members of the four perturbagen classes in the Touchstone database. Those classes are identified as follows: chemical compound/pharmacologic agent (CP); gene knock-down (KD); gene over-expression (OE); and perturbagen class (PCL). In the heatmap format, index signatures are compared with the entire Touchstone database of perturbagens (approximately 8400). Since our primary interest was to search for drugs and chemicals that had recognized mechanisms of action that might strengthen previous findings and support hypotheses for the therapeutic repurposing of drugs, we focused our searches in the detailed list format primarily on the CP agents.

The heatmap output that was presented gave the calculated median tau score (connectivity) for the 9 cell lines and a “summarization” measure. For all other results, the connectivity scores that were presented were the summarization measure. This measure was calculated across the 9 cell lines. The results are valuable since they provide a measure of the consistency of perturbations from one cell line to another. The algorithms for the calculation of tau and the summarization measure, and for the associated formulas, are presented at https://clue.io/connectopedia/cmap_algorithms (accessed on 29 July 2024). Gene-expression connectivity scores were also associated with a “rank” measure for that score; the rank score had an almost perfect non-linear inverse correlation with the gene expression similarity/connectivity score. These measures are detailed further in Figure S1. Thus, where “score” and “rank” are presented together in tables listing the gene-expression similarities between an index compound and a perturbagen in a particular PCL, the similarities of gene expression with a high score and low rank are the strongest (see Figure S1).

The results represent the status of the CLUE platform when accessed primarily between May, 2020 and July, 2023; the database is dynamic and changes slightly if new perturbagens are added.

### 2.2. CRS Assay Methodology

Female NSG-SGM3 mice [NOD.Cg-Prkdc^scid^ Il2rg^tmlWjl^ Tg(CMV-IL3,CSF2,KITLG) lEav/MloySzJ;stock #013062] were used for the study. The mice were ear notched for identification and housed in individually ventilated polysulfonate cages with HEPA filtered air at a density of up to 5 mice per cage. Cages were changed every two weeks. The animal room was lit entirely with artificial fluorescent lighting, with a controlled 12 h light/dark cycle (6 am to 6 pm light). The normal temperature and relative humidity ranges in the animal rooms were 20–26 °C and 30–70%, respectively. The animal rooms were set to have up to 15 air exchanges per hour. A 1% sucrose solution of filtered tap water, acidified to a pH of 2.5 to 3.0, and standard lab chow were provided ad libitum.

#### Study Design

1. Mice were irradiated and injected with PBMCs from a single donor on Day 0.
2. Animals were monitored for body weight and clinical observations once daily.
3. Animals were euthanized before study end if they showed >20% body weight loss or a body condition score of ≥2.
4. Treatment compounds were delivered in drinking water starting on Day 4 and continued for the duration of study.
5. Mice were dosed on Day 6 with monoclonal antibodies OKT3 or anti-CD28 to induce cytokine release [41], or with PBS as control.
6. Mice were bled via the retro-orbital (RO) method at 6 h post-dose on Day 6. Blood was processed and measured for human cytokines and chemokines using a MILLIPEX Human Cytokine/Chemokine/Growth Panel A Magnetic Bead Panel, Cat# HCYTOMAG-60K.
7. Body weight and clinical CRS score were assessed daily starting on Day 6. Mice were euthanized by CO_2_ asphyxiation at 72 h post-dose on Day 9.

The experimental design of the study is summarized by study day (SD) as follows: 
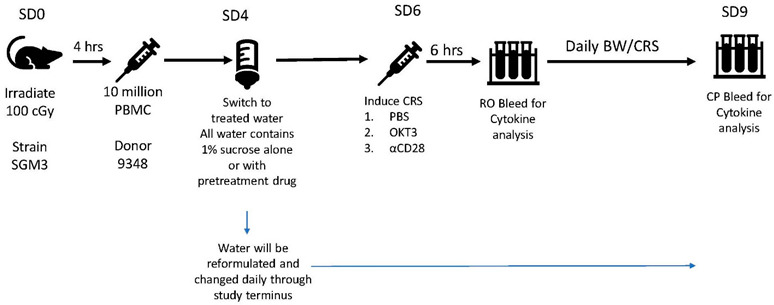


The clinical condition of the mice was rank scored as follows:

Clinical CRS score:

0: normal activity;

1: normal activity, piloerection, tiptoe gait;

2: hunched, reduced activity but still mobile;

3: hypomobile but mobile when prompted;

4: moribund (non-responsive to touch).

Complete details of the animal experiments are presented in Table 2.

Levalbuterol hydrochloride, albuterol sulfate, and phenoxybenzamine hydrochloride were purchased as reference standards from the United States Pharmacopeia. Arformoterol tartrate and theophylline (anhydrous powder) were purchased from Sigma-Aldrich Chemicl Company.

### 2.3. Statistics

Group sizes of these valued experimental animals were kept as small as possible and were consistent with the spirit of FDA Amendment 2.0. The cytokine and chemokine data from these small groups largely failed normality distributions. Thus, all analyses were conducted with the Kruskal–Wallis multiple comparative approach for nonparametric data followed by post-hoc adjustment of *p* values with Dunnett’s test. Each drug treatment effect was compared only with its sucrose control. A *p* value of 0.05 (*) or less was considered statistically significant; ** *p* < 0.01; *** *p* < 0.001; **** *p* < 0.0001.

## 3. Results

### 3.1. Analyses of Gene Expression Signatures of β2-Adrenergic Agonists and Related Compounds in the Harvard/MIT Broad Institute Genomic Database, CLUE

In preliminary analyses, salbutamol (albuterol), the racemic mixture of the drug that was prominent in the Norwegian and Israeli epidemiological studies in relation to PD frequency [5,18] was profiled in CLUE. Salbutamol showed significant connectivity with several beta-adrenergic receptor agonists when profiled in CLUE; 8 had gene-expression connectivity scores that were higher than 90.0 in relation to salbutamol (Table S1).

Also, when CLUE was queried for connectivity with dopamine receptor agonists, 5 had high scores in relation to salbutamol; these included dopamine itself (Table S2). In view of the apparent associations between epinephrine and PD, it would appear to be important that there is an overlap of the agonist activity of epinephrine on β2AR and dopamine receptors [46,47].

**Figure 1:**
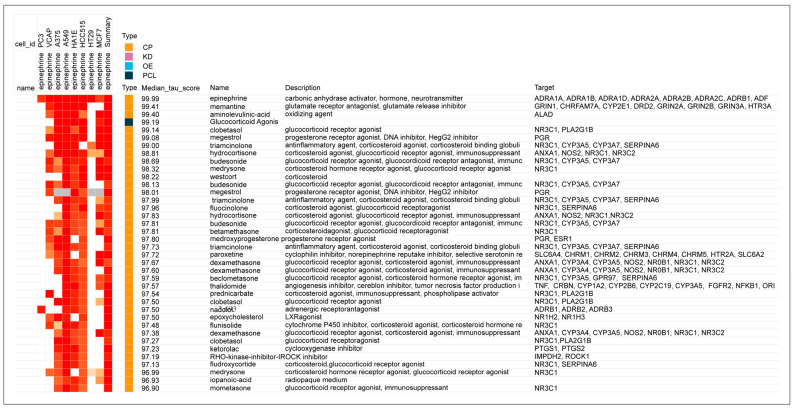
Heat map of association strengths (median tau score) between gene-expression signatures of epinephrine in 8 malignant cell cultures and those for all perturbagen entries in the Touchstone database, with targets. Perturbagen classes: Chemical compound (CP); gene knock-down (KD); gene over-expression (OE); and perturbagen class (PCL).

**Figure 2:**
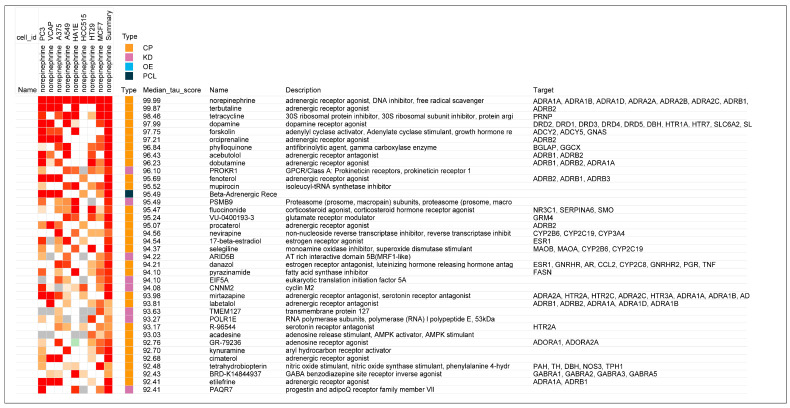
Heat map of association strengths (median tau score) between gene-expression signatures of norepinephrine in 8 malignant cell cultures and those for all perturbagen entries in the Touchstone database. Perturbagen classes: Chemical/pharmacologic agent (CP); gene knock-down (KD); gene over-expression (OE); and perturbagen class (PCL).

The potential anti-inflammatory importance of β2-adrenergic agonists was first observed in the current study with the observation of a remarkable similarity of the gene-expression signatures of epinephrine to those of glucocorticoid receptor (GR) agonists. A portion of the heat map with the highest tau scores when epinephrine was modeled in CLUE already showed the predominance of GR agonists with high tau scores for the top matches in the database; the protein targets of the matching compounds were also replete with the codes, NRC31, glucocorticoid receptor, and NRC32, mineralocorticoid receptor (Figure 1). In comparison, norepinephrine, which has approximately equal β1-adrenergic activity as epinephrine but has little or no β2 activity, showed essentially no connectivity to GR agonists in a comparable portion of its heat map (Figure 2). More than 60% of the gene-expression signatures with connectivity to epinephrine in Figure 1 were GR agonists, while there was only one (0.025%) GR agonist with a high tau score that matched with norepinephrine (Figure 2).

The detailed analysis outputs from the CLUE platform added further evidence of the strong association of epinephrine with drugs that have GR receptor agonist activity. A table is presented that shows the gene enrichment scores for CMap classes in the Touchstone database (Table 3). A high gene enrichment score was noted for the 44 GR agonists in this CMap class. As would be expected, there was also a high enrichment score for the 11 beta-adrenergic receptor agonists in this CMap class (Table 3). Consistent with the almost complete lack of GR receptor agonists in the norepinephrine heat map (Figure 2), no connections with the GR CMap class were found in the comparable CMap class output for norepinephrine (Table 4). However, the general category of beta-adrenergic receptor agonists is represented (Table 4) because epinephrine and norepinephrine have equal β1-adrenergic potency.

Still further evidence that an association exists between GR receptor agonist activity and the neurotransmitter epinephrine, and that this is unique to this agent that has β2-adrenergic activity and not shared by norepinephrine, was observed in a comparison of the detailed analyses associated with the CMap class score differences. The strengths of associations in gene signatures with GR receptor agonists for epinephrine and norepinephrine are presented in Table 5 and Table 6, respectively. Of the 44 glucocorticoids in the CMap class, all had gene-signature connectivity scores above 90.0 in relation to epinephrine (Table 5), while only 7 had scores above 90.0 for connectivity with norepinephrine and some had negative (opposing) connectivity (Table 6). (As discussed in the Methods section and explained further in Figure S1, the gene-expression connectivity scores in Table 5 and Table 6 are associated with a “rank” that is essentially an inverse measure to the gene-expression “score”).

**Figure 3:**
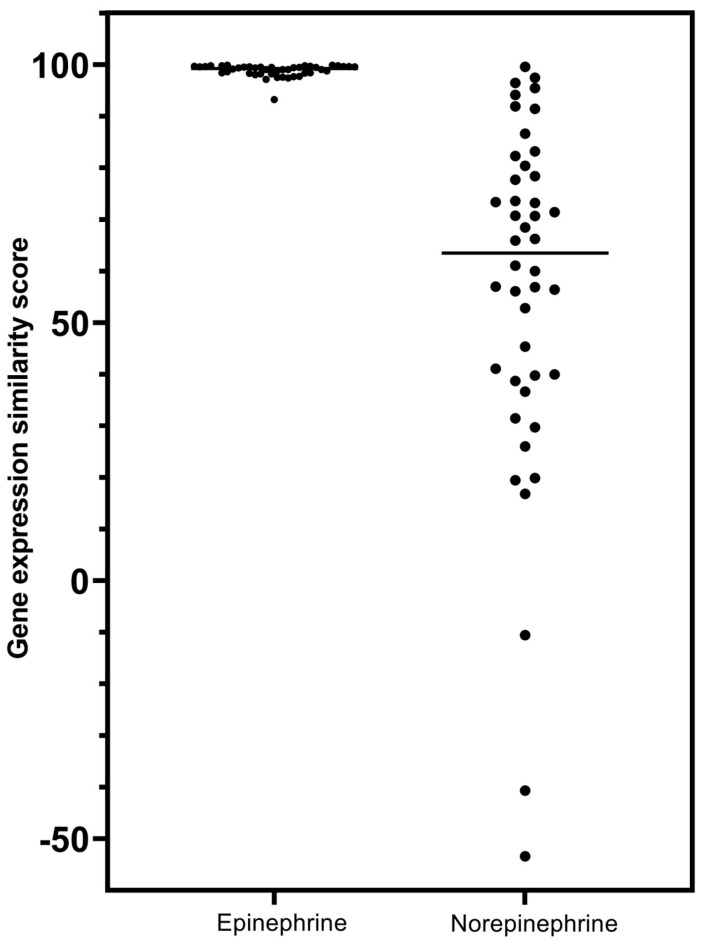
Comparison of epinephrine and norepinephrine gene-expression scores as glucocorticoid receptor agonists. The horizontal lines are the median values for the individual distributions. Mann–Whitney analysis of the distributions showed a statistically significant difference (*p* < 0.0001), with strong association for epinephrine with gene-expression similarity to glucocorticoid agonists.

The gene-expression score rankings of glucocorticoid receptor agonists for epinephrine and norepinephrine are presented graphically in Figure 3.

It is of particular interest, and was unexpected, that the perturbagen class output for epinephrine (Table 3) included leucine rich repeat kinase (LRRK) inhibitors showing gene-expression connectivity to epinephrine with a strong positive score. Among the agents in the CLUE database, LRRK2 inhibitors and XMD-1150, Broad Institute ID (BRD)-K01436366 and XMD-885 (BRD-K64857848), ranked with connectivity scores to epinephrine of 90.78 and 90.47, respectively. LRRK2 inhibitors are currently a subject of major focus as potential treatments for PD [48,49,50,51]. Table 3 also includes a positive score for gene-expression connectivity with epinephrine for vascular endothelial growth factor receptor (VEGFR) inhibitors; this is commented upon below. Quite remarkably, the CLUE output in Table 3 also includes a positive score for connectivity of epinephrine with rapidly accelerated fibrosarcoma (RAF) inhibitors. The proto-oncogene *B-RAF* is part of the pathway of *RAF* activation of many cancers [52]. Although this could be of considerable interest in relation to possible anti-neoplastic activity of β2AR agonists, it was not pursued in the current study.

It should be noted that the positive rankings for LRRK2, VEGFR, and RAF inhibitors in relation to epinephrine were not identified with gene-expression connectivity to norepinephrine (Table 4), which is consistent with the association of these properties with β2-adrenergic activity.

The CLUE analyses of a marked difference between the associations of epinephrine and norepinephrine with gene signatures that are predictive of GR agonist activity parallelled the established biological differences between the agonist activities of epinephrine and norepinephrine (Table 7). Epinephrine and norepinephrine have essentially equal α1-, α2-, and β1-adrenergic agonist activity, but epinephrine is substantially more active than norepinephrine as a β2-adrenergic agonist. The evidence outlined above that supports an anti-inflammatory activity of β2-adrenergic agonists, and a potentially therapeutic effect in relation to the neuroinflammation aspect of PD, is a major focus of these investigations. Also, the fact that GR agonists are widely recognized for their anti-inflammatory activity adds to the plausibility of these associations with β2-adrenergic activity.

When the natural endogenous human glucocorticoid cortisol (hydrocortisone) was profiled in CLUE, as expected, it showed strong gene-signature connectivity with the synthetic glucocorticoid drugs in the CLUE CMap class (Table 8). Interestingly, it also ranked the in the beta-adrenergic receptor agonist class with a high enrichment score, similar to that for epinephrine (Table 3). The detailed analyses also demonstrated that the 44 synthetic glucocorticoids in the CMap class all had gene-signature connectivity scores above 90.0 with hydrocortisone (Table 9); again, this is essentially identical to the findings with epinephrine (Table 5).

**Figure 4:**
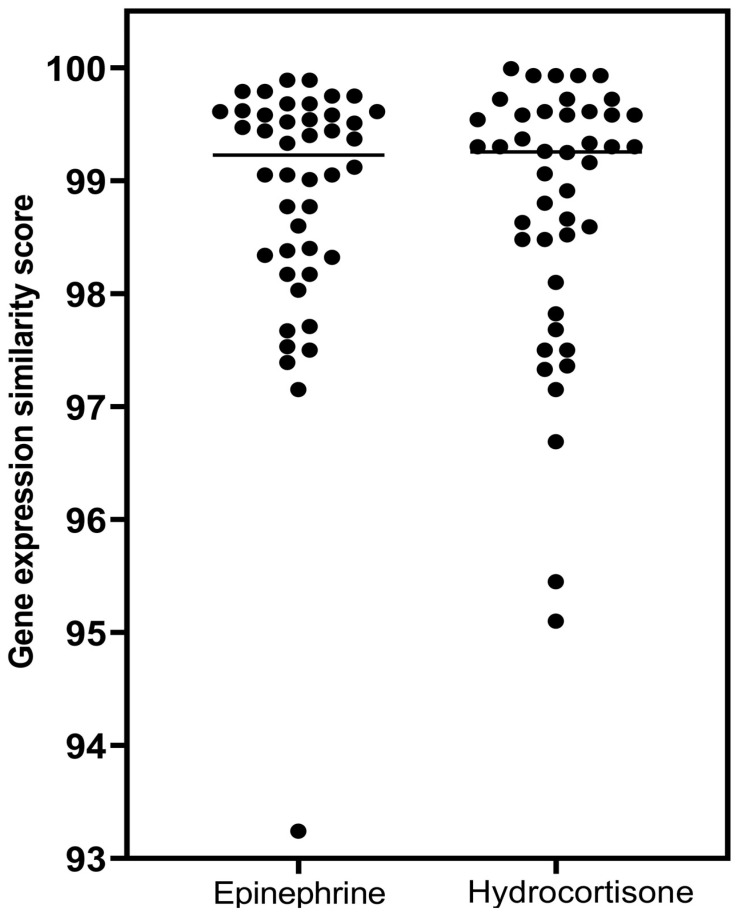
Comparison of epinephrine and hydrocortisone gene-expression scores as glucocorticoid receptor agonists.

To summarize, Figure 4 presents the comparison between the gene-expression signatures when CLUE was probed with epinephrine and hydrocortisone as index compounds (i.e., Table 5 vs. Table 9 results).

The horizontal lines represent the median values for the individual distributions, which were essentially identical. Mann–Whitney analysis of the distributions showed no difference in gene-expression characteristics for epinephrine and hydrocortisone as glucocorticoid receptor agonists (*p* = 0.805).

The gene-product targets for the 44 glucocorticoid analyses in this CMap class are presented in Table S4. All members of this class have the glucocorticoid receptor, NR3C1, as a target; some also have the mineralocorticoid receptor, NR3C2, as a target. Only the natural human glucocorticoid cortisol (hydrocortisone) and the synthetic drugs dexamethasone and amcinonide have the gene product ANXA1 as a target. Annexin A1, also termed lipocortin I, is the product of the gene *ANXA1*, and appears to primarily serve the anti-inflammatory effect of several glucocorticoids [53]. In addition to the gene-product targets for the glucocorticoids, Table S4 includes the Broad Institute ID numbers for each analysis of gene expression in the database.

It should be noted that the CMap class output for hydrocortisone (Table 8) included a strong group score (92.61) for gene-expression connectivity to mitogen-activated protein kinase kinase (MEK) inhibitors. This kinase is on the pathway signaling malignant proliferation and survival, i.e., RAF > MEK > extracellular signal-related kinases (ERK). This is similar to the observations noted above for epinephrine (Table 3), where gene-expression connectivity was observed for inhibitory mediators of the anti-proliferative/anti-neoplastic effects of LRRK2, VEGFR, and RAF. In the case of the MEK inhibitors, the drug with the highest score for the class when probed separately in CLUE was selumetinib (96.51), which has been proven to have a broad role as an anti-neoplastic agent [54,55].

The striking similarities in the gene-expression signature connectivity for epinephrine and hydrocortisone drew attention to their known interrelationships in regard to the synthesis of cAMP [42,56,57]. Perez [42] noted that α2-adrenergic agonist activity mediated decreased synthesis of cAMP. When the classical non-competitive, irreversible α2-adrenergic antagonist phenoxybenzamine was modeled in CLUE, it also showed considerable gene-expression signature connectivity with GR agonists. The gene enrichment score for phenoxybenzamine with the GR agonist CMap class is shown in Table 10.

Detailed analysis of the gene-expression connectivity scores for phenoxybenzamine with GR receptor agonists is listed in Table S3.

The strong gene-expression connectivity of phenoxybenzamine with GR agonist activity and the potential anti-neoplastic properties of phenoxybenzamine (Table 10) have been presented in previous reports [43,44].

The associations between agents that increase cAMP levels and GR receptor agonist activity were investigated further by modeling two methylxanthine drugs, theophylline and caffeine, in CLUE. Theophylline has a long therapeutic history as a bronchodilator in the treatment of respiratory diseases. As discussed above, it does not directly increase synthesis of cAMP but results in higher levels of cellular cAMP by inhibition of phosphodiesterase enzymes that metabolize cAMP. Caffeine has similar activity but is a much more potent CNS stimulant than a peripheral agonist.

The CMap class output scores from CLUE for theophylline are presented in Table 11. Gene-expression connectivity scores vs. GR agonists are presented in Table S5.

The CMap class output scores from CLUE for caffeine are presented in Table 12. The GR agonist class scored substantially lower for caffeine than for theophylline. Gene-expression connectivity scores vs. GR agonists are presented in Table S6.

**Figure 5:**
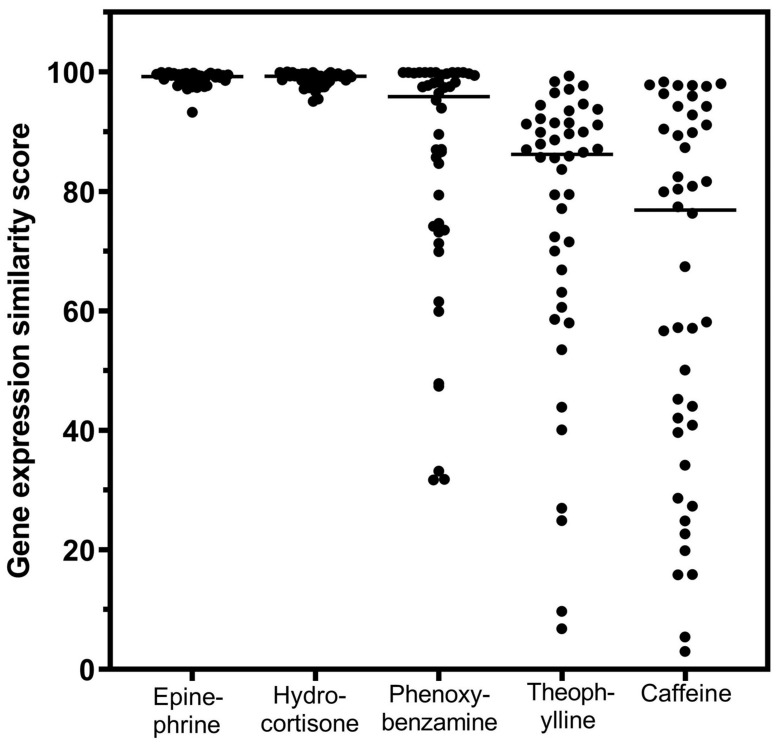
Comparison of beta2-adrenergic-related agents and hydrocortisone for gene-expression connectivity scores as glucocorticoid receptor agonists. The horizontal lines are the median values for the individual distributions. The relative differences among the gene-expression scores can be appreciated visually. Caffeine was not studied further.

A summary of agents that were investigated in relation to their gene-expression signatures with connectivity to GR agonists is presented in Figure 5.

Epinephrine, a potent β2AR agonist, has been presented as a prototype for drugs of this class in these studies because levalbuterol and arformoterol have not been profiled for gene-expression connectivity in CLUE. In July 2020, as our interest in levalbuterol advanced, we formally nominated it to the Broad Institute for gene-expression connectivity study in the CLUE database, based on its apparent anti-inflammatory potency [7]; it remained in the queue for possible testing for several months but was never screened.

It would appear to be significant that the drugs noted in Figure 5 that have gene-expression signatures with connectivity to GR agonists are all capable of mediating an increase in cellular cAMP. Since increases in cAMP are characteristic of drugs that mediate anti-inflammatory effects, it appears that this may represent a unifying hypothesis for this study.

### 3.2. CRS Assays of Agents with β2-Adrenergic and Related Activities

The CRS assay was used to test the efficacy of the several β2-adrenergic-related drugs discussed above for possible relationships between predictions generated in the CLUE genomic analyses and a biological test of anti-inflammatory activity in the humanized mouse assay. All animal assays were carried out in the Jackson Laboratories facility in Sacramento, California.

Several of the drugs of interest were tested for their ability to modulate cytokine release in vivo after the mice were challenged with injections of the monoclonal antibodies OKT3 or aCD28. The application of an in vivo model to simulate a clinical translation was considered a feature of these investigations. The drugs were administered in the drinking water. They are all water soluble and are known to have good bioavailability by the oral route. Doses were calculated from the standard human clinical range of mg/kg body weight per day. An FDA factor of 12.3 was applied for conversion from human to mouse mg/kg dose. A mouse body weight of 20 g and an average intake of drinking water of 4 mL/day were assumed. The resulting doses were as follows: albuterol sulfate, 8 µg/mL drinking water; levalbuterol HCl, 4 µg/mL; phenoxybenzamine HCl, 5 µg/mL; theophylline, 400 µg/mL; and arformoterol tartrate, 0.008 µg/mL.

**Figure 6:**
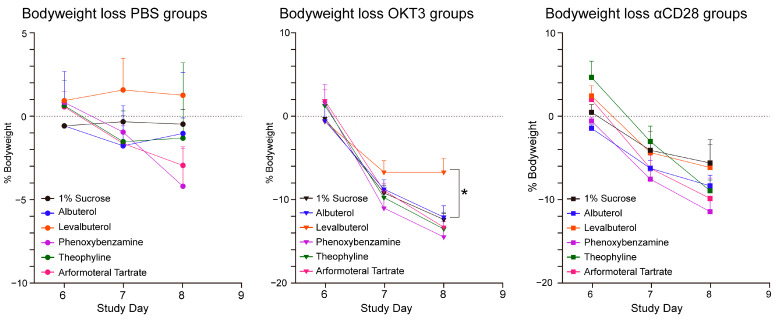
Percent bodyweight loss calculated from each animal’s initial body weight at Study Day 0.

Mice treated with levalbuterol showed statistically significant lower bodyweight loss than the control group (1% sucrose) when CRS was induced with OKT3 (Figure 6).

**Figure 7:**
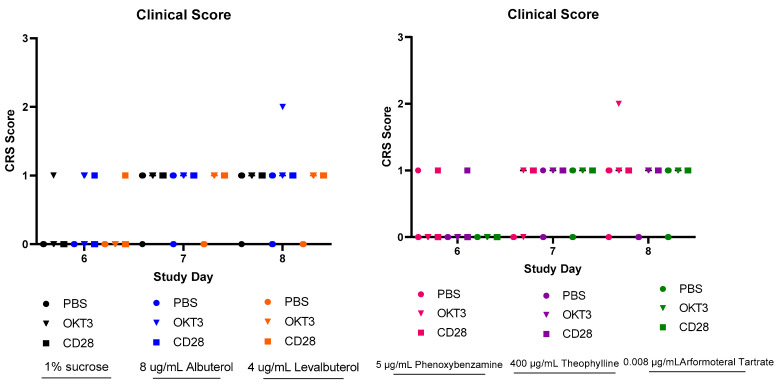
CRS clinical scores for the period following toxic induction of cytokine release with OKT3 or aCD28.

There were no statistically significant differences in the clinical scoring between any of the groups and the control group (Figure 7). All 90 animals included in the study survived to the time of blood collection by cardiac puncture on Study Day 9, 72 h after toxic induction with OKT3 or aCD28. This would suggest that the doses translated from human to mouse were reasonably appropriate.

There were no statistically significant differences in the clinical scores that were attributable to the pretreatments; the doses of pretreatments were calculated from typical human doses that were translated using FDA factors.

**Figure 8:**
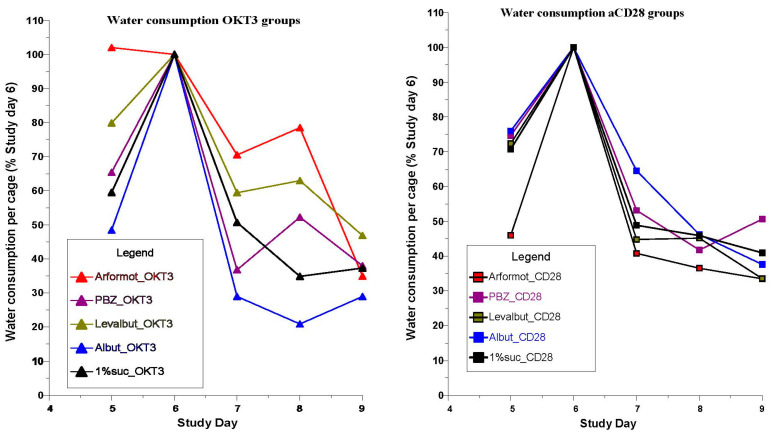
Water consumption recorded per cage of 5 mice as a percentage of the intake on Study Day 6 prior to the induction challenges with OKT3 and anti-CD28.

Water consumption in both the OKT3 and anti-CD28 groups peaked on Study Day 6 before toxicity was induced and declined progressively to Study Day 9 (Figure 8). Analysis of the data was limited since water consumption was recorded per cage for each group of five mice. However, one individual comparison (Wilcoxon signed-rank test) for water consumption for levalbuterol vs. sucrose control in the OKT3 groups did indicate a statistical advantage favoring more water intake for levalbuterol (*p* = 0.068). (Data for the theophylline groups were accidentally not recorded).

The toxic antibody challenges to induce cytokine release caused marked decreases in water consumption in all of the groups. Since the pretreatment drugs were administered in the drinking water, it would be expected that drug delivery was compromised.

**Figure 9:**
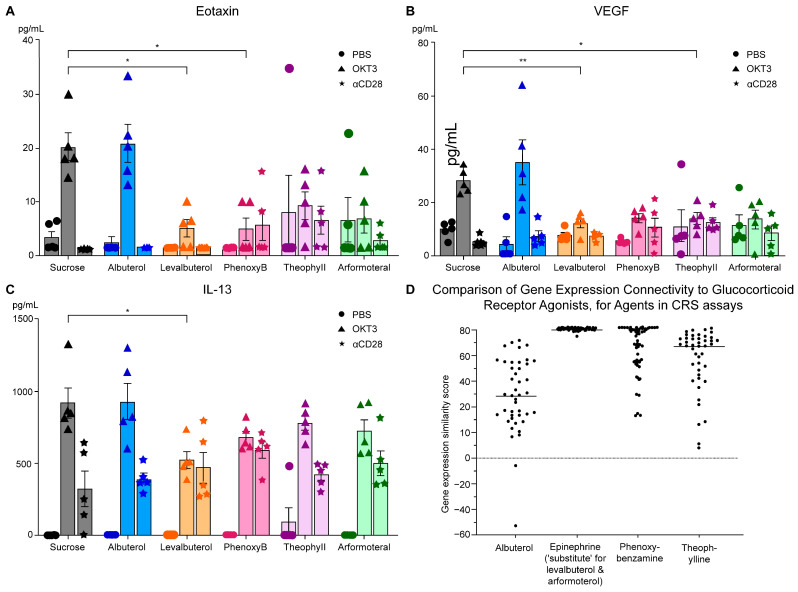
The effects of β2-adrenergic-related drugs on the release of eotaxin, VEGF, and IL-13.

Analyses of the effects of the beta2-adrenergic agonists and related compounds on the release of cytokines and chemokines 6 h after induction with OKT3 in the CRS assay demonstrated the following primary outcomes. Figure 9 presents the results for the inhibition of the release of the eosinophil-selective chemokine eotaxin, which is responsible for a number of eosinophilic disorders, and the angiogenic proto-oncogene vascular endothelial growth factor (VEGF), by several of the β2-adrenergic related drugs. Albuterol, the racemic mixture of the levo and dextro isomers, was not different from the sucrose control in any of the assays. It should be noted that albuterol was administered at twice the concentration of levalbuterol; therefore, the mice received the same dose of the levo isomer as those receiving the pure levalbuterol. However, as noted above, the dextro isomer is pro-inflammatory and interferes with the anti-inflammatory activity of the levo isomer. Levalbuterol also inhibited the release of the pro-inflammatory cytokine IL-13 with OKT3 induction (Figure 9). The relationships between the treatment drugs and their gene-expression connectivity to glucocorticoid receptor agonist activity are also included in Figure 9.

Figure 9 elaborates the results for eotaxin (A), VEGF (B), and IL-13 (C) in the CRS assay. In Figure 9D, comparisons are illustrated in the same order for the gene-expression connectivity of the agents as glucocorticoid receptor agonists in the CLUE database. As noted, the gene-expression connectivity for epinephrine, the prototypical beta2-adrenergic agonist, was substituted for levalbuterol and arfomoterol since these drugs were never profiled in the CLUE database. Albuterol, the racemic mixture of R and S levalbuterol, showed no inhibition of cytokine/chemokine release in any of the assays (A, B, C).

The changes seen in cytokine and chemokine inhibition at 6 h after OKT3 induction did not persist until assays on Day 9, 72 h after induction. A possible explanation may be related to the fact that water intake containing the adrenergic-related pretreatments was reduced by approximately 55 to 70 percent among the treatments by Day 9 (Figure 8).

**Figure 10:**
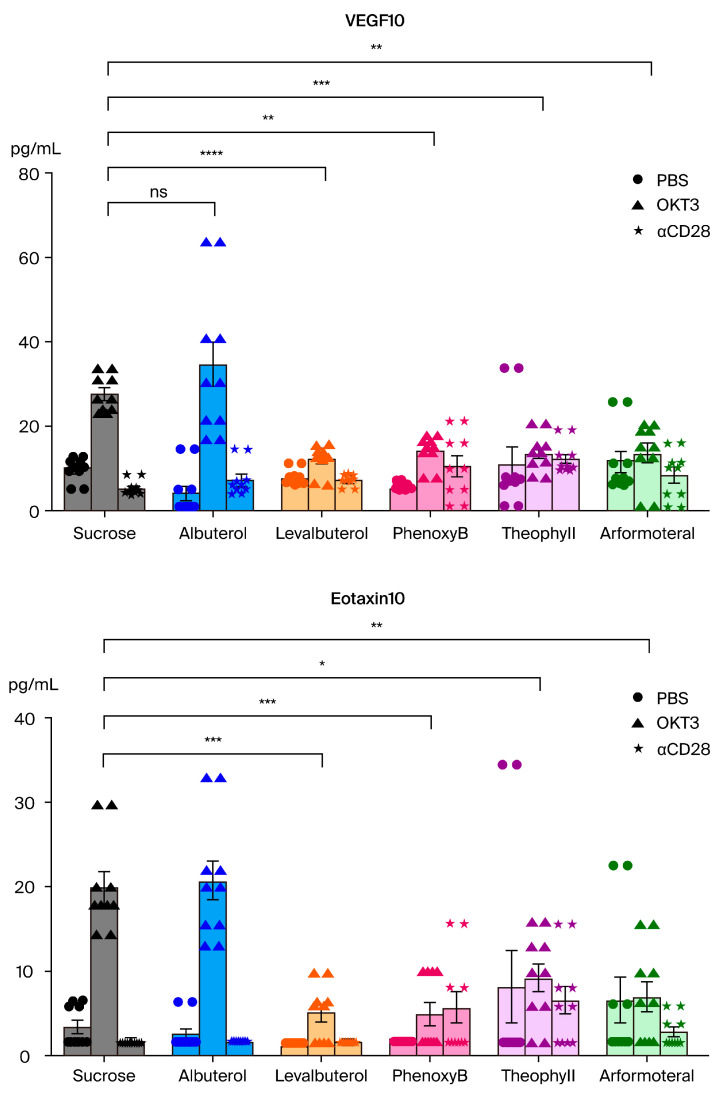
“Power analysis” of the results for the VEGF and eotaxin assays.

The results for eotaxin and VEGF with levalbuterol (Figure 9) showed that some of the other beta2-adrenergic-related drugs were quite close to statistical difference from their controls, so a simple “power analysis” was conducted by duplicating the data to double the sample size to 10 mice/group; the variance was kept the same. All the drug treatments except albuterol showed significantly less elaboration of VEGF and eotaxin with these increases in sample size (Figure 10).

The data were duplicated from the five-animal CRS assays but the variance remained unchanged.

Possible trends toward statistical significance were examined for all the cytokines and chemokines that were measured. Unpaired t-tests, without correction for multiple comparisons, identified the following inhibitory effects of levalbuterol on cytokine elaboration after OKT3 induction: TNFα, *p* = 0.0321; TNFβ, *p* = 0.0373; M-CSF1, *p* = 0.0342; IL-1α, 0.0399; and IL-18, *p* = 0.0003.

The MILLIPLEX panel used to quantify cytokines in the above assays identified eotaxin-1 (CCL11), which is the most extensively characterized and studied of the eotaxins. When eotaxin links to its most specific receptor, CCR3, in organs and the brain, it recruits eosinophils that upon degranulation release eosinophil granule proteins, growth factors, and cytokines causing cellular damage; eotaxin plays a central role in mediating a number of eosinophilic disorders, including atopic dermatitis, eosinophilia in asthma, chronic rhinosinusitis, and eosinophilic esophagitis [58,59]. Teixeira et al. [60] reviewed the association of increased blood levels of eotaxin-1/CCL11 in patients with major psychiatric disorders including schizophrenia, major depression, bipolar disease, autism spectrum disorder, dysthymia, and Alzheimer’s disease. They also discussed the presence of the CCR3 receptor on microglia, and Peterson et al. [11] have proposed a pro-inflammatory role for activated microglia in the destruction of dopaminergic neurons in PD. Chandra et al. demonstrated that eotaxin was markedly elevated in the substantia nigra pars compacta in post-mortem brains of PD patients compared with non-affected controls [61].

The finding that IL-13 release was also inhibited by levalbuterol (Figure 9) is of interest because IL-13 is one of the cytokines that is central to cellular trafficking; blocking its ligands is a feature of dupilumab in the treatment of chronic obstructive pulmonary disease (COPD) and other eosinophilic disorders [59,62].

Figure 9 also presents the results for the inhibition of the release of VEGF by levalbuterol and theophylline. There is a vast body of literature on the importance of VEGF in neoplastic disease, including an earlier comprehensive review by Goel and Mercurio [63], and recent efforts to block certain particularly aggressive cancers depend on the binding of VEGF to a co-factor, neuropillin-2 [64].

The MILLIPLEX panel that was used to quantify this cytokine in the CRS assays was specific for the VEGF_165_ variant. Although it is many decades since the recognition of the involvement of VEGF in tumor angiogenesis, treatment complexity persists. Mabeta and Steenkamp [65] presented a recent review of VEGF variants; VEGF_165_ is the prototypical cytokine and is upregulated in approximately 70% of tumors including breast cancer, glioblastoma multiforme, ovarian cancer, melanoma, esophageal cancer, gastrointestinal tract cancers, lung cancer, renal carcinomas, and others. VEGF_165_ promotes sprouting of existing vessels as well as splitting of vessels as part of its angiogenic effects; these processes may be part of an effort by tumors to escape therapeutic interventions that produce conditions jeopardizing tumor expansion [65]. It is interesting to speculate that inhibition of release of VEGF, as seen in Figure 9, may be superior to the complexities of control of angiogenesis at the tumor level.

The principal experimental findings of this investigation may be summarized as follows:

(a) The prototypical beta2-adrenergic agonist epinephrine, but not norepinephrine (minimal beta2-adrenergic activity), showed prominent similarity (connectivity) to the gene-expression of glucocorticoid receptor agonists when modeled in the Harvard/MIT genomic database, CLUE. This was seen consistently and progressively in the three data outputs from this cloud-based software, including a heat map of individual assays with eight malignant tumor cultures, cumulative scoring of gene-expression among the eight cultures for similarity to glucocorticoid receptor agonists, and the individual scores and ranks for epinephrine and norepinephrine gene expression for each member of the glucocorticoid class in the CLUE database;
(b) The association of beta2-adrenergic activity with glucocorticoids suggested potential anti-inflammatory activity for these drugs and possible value in suppression of the neuroinflammation that is characteristic of the progression of PD and other neurodegenerative disorders. Several beta2-adrenergic agonists and adrenergic-related drugs were tested for anti-inflammatory activity in mice engrafted with human PBMCs. Pretreatment with these drugs prior to challenge with monoclonal antibodies known to result in activation and release of cytokines and chemokines inhibited elaboration of eotaxin-1/CCL11, VEGF_165_, and IL-13 to varying extents.

## 4. Discussion

### 4.1. Absence of Classical Glucocorticoid Toxicity

The marked similarity between gene-expression connectivity for epinephrine (and presumably other β2AR agonists) and glucocorticoids, as presented in these investigations, raises the question of why β2AR agonists have shown absolutely no evidence of the classical adverse effects of glucocorticoids. This is true with chronic treatment with β2AR agonists at pharmacological doses in their primary role as bronchodilators. Adverse side effects of glucocorticoids include hypertension, hyperglycemia, osteoporosis, glaucoma, cataract formation, peptic ulcers, gastrointestinal bleeding, and others [66]. These effects are dose-dependent and reduction in dosage leads to loss of therapeutic effectiveness.

Dose limitations represent a major disadvantage in the clinical use of glucocorticoids. In addition to their efficacy as anti-inflammatory/immunomodulatory agents, these drugs have major anti-neoplastic effects in the treatment of several hematopoietic malignancies with lymphatic lineage, including chronic lymphocytic leukemia, acute lymphoblastic leukemia, multiple myeloma, Hodgkin’s lymphoma, and non-Hodgkin’s lymphoma [67,68]. Induction of apoptosis appears to be a primary mechanism in the treatment of these malignancies [69].

There has been considerable effort directed in attempts to separate the desired effects of glucocorticoids from the initiation of adverse events. Agents of this type are chemically non-steroidal and have been variously termed as “dissociated glucocorticoid receptor ligands” [70], “selective glucocorticoid receptor agonists” (SEGRAs) [71], or examples of “biased signaling” [72]. Kleiman and Tuckerman summarized the results of some of the biological trials with SEGRAs, which have shown limited success [73].

The fact that epinephrine, the prime example of a β2AR agonist, showed exactly the same gene-expression profile as hydrocortisone in the CLUE analyses (Table 5 and Table 9; Figure 4) but was not associated with any of the adverse effects of glucocorticoids is now known with confidence to be related to the gene-signaling pathways from different receptor ligands. The biological result is related to which gene-signaling pathways are favored [74].

An updated comprehensive review of the glucocorticoid receptor by Nicolaides et al. [75] discusses the two major classifications of the signaling of gene expression in relation to the potential for adverse events with longer treatment and higher doses versus the pathway to therapeutic predominance. The contrasting pathways are termed “transactivation” and “transrepression.” Transactivation correlates with downstream transcription of protein sequences that mediate the toxic manifestations of glucocorticoid treatment (diabetes mellitus, osteoporosis, hypertension, etc.). Transrepression is primarily associated with the therapeutic effects of glucocorticoids, i.e., their anti-inflammatory, immunomodulatory, and certain anti-neoplastic effects. The mechanisms of transactivation and transrepression are complex and have been discussed in extensive detail by Lesovaya et al. [76]. In view of the potential anti-inflammatory effects of β2AR-related drugs observed in the present study, it would be expected that they mediate via a transrepression effect at the GR.

The chapter by Nicolaides et al. [75] also discusses the evolution of the glucocorticoid receptor, which apparently represents an important step in the survival advantage of a branch that ultimately led to mammalian species. That step, which included specificity of the GR for cortisol, took place about 450 million years ago with the first appearance of the GR in ray-finned fish and then in land vertebrates. Of interest to the present study, the evolution of the sympathetic nervous system took place in that same period of geologic time, and it is presumed that a sympathetic nervous system represented a level of functional sophistication that contributed to survival to further evolution [77].

The adrenal steroids and sympathetic nervous systems are critical parts of the integrated response to stress in humans today and this may be the reason that the CLUE platform of the Harvard/MIT Broad Institute database showed essentially the same gene-expression signatures for hydrocortisone and epinephrine, as noted above. In these integrated systems, glucocorticoids have been demonstrated to promptly increase elaboration of cAMP by several mechanisms that would be expected to inhibit the release of pro-inflammatory cytokines and chemokines: (a) hydrocortisone (cortisol) directly activates adenylyl cyclase to elaborate cAMP; (b) it increases the density of β2ARs on cell membranes within elements of the innate immune system; (c) it facilitates linking of those receptors to adenylyl cyclase to increase cAMP; (d) it preserves responsiveness (i.e., reverses tachyphylaxis) that may develop with continuous occupancy of β2ARs [56,57,78,79,80,81,82]; and finally, (e) there is the added fact that, as part of the stress response, hydrocortisone selectively activates the phenylethanolamine N-methyltransferease (PNMT) enzyme in the adrenal medulla to increase the synthesis of epinephrine [75,83,84]. It is interesting to speculate that the selective beta2-adrenergic-related agents studied in this investigation may extend the clinically therapeutic applications of the glucocorticoid–sympathetic stress response without incurring the adverse effects of higher glucocorticoid doses.

### 4.2. Continued Focus on Exercise as PD Therapy

The association of exercise with elaboration of the beta2-adrenergic agonist epinephrine, as discussed in detail in the Introduction to this investigation, appears to be of continued relevance in the treatment of PD.

Dolhun et al. have published an extensive compilation of experiences from the Michael J. Fox Foundation with patients from the community that have engaged in various exercise regimens to treat or delay progression of their PD pathology [85]. Many of the regimens are of an extreme nature compared with classical physical therapy sessions and speak to the question of whether it takes this level of activity to accomplish more significant clinical results. The manual was published on 2 January 2024 and can be downloaded with the following link: “Exercise_Guide_Singles_02.01.24”.

Chauquet et al. found that exercise on a running wheel reversed ageing phenomena in the microglia of mice at 18 months of age [86]. Gene expression of microglia with exercise reverted to that of young mice and demonstrated a stimulation of hippocampal neurogenesis.

Augusto-Oliveira et al. reviewed the effects of exercise as a “holistic” therapy that impacts upon the brain through multiple mechanisms that contribute to the development and improvement of cognitive capacity and mitigation of age-related cognitive decline [87]. These effects of exercise are primarily mediated by a group of growth factors, neurotrophins. Four of these factors have been extensively characterized, including nerve growth factor (NGF), with a role for sensory and sympathetic neuron integrity; brain-derived neurotrophic factor (BDNF), critical for learning and memory through neurogenesis in the hippocampus, cerebellum, basal forebrain, and cortex; neurotrophin-3 (NT-3), which functions as a growth factor in both the central nervous system and the periphery with activation of tyrosine kinase receptors TrkC and TrkB, which are essential for adequate proprioception; and neurotropin-4 (NT-4), which also activates tyrosine kinase receptor TrkB.

### 4.3. Levalbuterol and Tyrosine Hydroxylase

In particularly interesting observations by Flydal et al. [88], levalbuterol was identified as a small molecular “chaperone” that stabilized the enzyme tyrosine hydroxylase. This enzyme catalyzes the rate-limiting step in the synthesis of dopamine. It is depleted or dysfunctional in tyrosine hydroxylase deficiency syndrome and in PD. In an effort to repurpose treatments for these deficiencies, the investigators screened 1280 recognized drugs for their abilities to prevent excessive aggregation and/or misfolding of the tyrosine hydroxylase molecule and to reduce the feedback inhibitory effects of dopamine on the activity of the enzyme. Through active site modeling, levalbuterol was proposed to bind to the same iron moiety as dopamine at the active site. However, binding by levalbuterol did not inhibit activity but did competitively reduce the feedback-inhibitory effects of dopamine. These properties of levalbuterol may have value in reducing the dyskinesias associated with the chronic treatment of PD. Nineteen drugs from the initial screening showed promise, but validation studies eliminated all but four. Of those, levalbuterol demonstrated dose-related efficacy most consistently [88].

### 4.4. Clinical Translation of the Findings

The drugs studied in the present work are all FDA-approved medications, which could facilitate their possible translation to therapies that have relevance to neurodegenerative and neoplastic disorders. There are considerable clinical data regarding the use of levalbuterol and other beta2-adrenergic selective agents for the treatment of bronchial asthma in children and adults. The doses that were used in the present studies to test their effects on cytokine release in the human PBMC-engrafted mice were translated from human adult doses that relieve asthmatic attacks. Heart rate changes were not monitored in the mice but there were no untoward effects noted. In a clinical comparison between levalbuterol and the racemic drug albuterol in children aged 4 to 11 years, levalbuterol appeared to be superior in terms of improvement in respiratory function indices [89]. Children who received the recommended dose of levalbuterol for this age group (0.31 mg by nebulized inhalation) showed no mean change in heart rate; those that required double this dose for satisfactory treatment of an asthmatic attack had a mean increase in heart rate of approximately seven beats per minute. It must be noted that this route of administration essentially delivers the entire dose to the lungs where there is rapid systemic absorption that impacts the heart; this would not be the case if the drug was administered episodically by the oral route. It was administered continuously in drinking water in the mouse studies. A recent meta-analysis covering the use of this class of drugs from 1996–2021 demonstrated their safety [90].

Clinical translation would also be aided by the fact that there are no formulation issues for levalbuterol. There are three FDA-approved dose levels that could be used for episodic oral administration.

### 4.5. Limitations

An unfortunate limitation in the studies noted above was the fact that levalbuterol and arformoterol have never been profiled for gene-expression connectivity to glucocorticoids by the Harvard/MIT Broad Institute. We did formally nominate them for assay in CLUE, but this has not taken place. This remains to be addressed in future studies. Another limitation was that we were not able to assay the proto-typical beta2-adrenergic agonist epinephrine in the mouse CRS assay. The assay was designed to provide low-stress delivery of pretreatments to the mice by providing the drugs in the drinking water. One concern is that epinephrine may have undergone autoxidation under these conditions.

### 4.6. Future Studies

It is still considered extremely desirable to attempt a study of epinephrine via the mouse CRS assay. One approach may be to deliver epinephrine in an osmotic pump as hydrochloride salt; it should be stable and protected from autoxidation in this form. Also, Jackson Laboratories have several mouse strains of PD variants that could possibly provide models for testing efficacy in PD with epinephrine delivered in chronic administration by osmotic pump.

This same approach can be utilized to test the anti-neoplastic efficacy of epinephrine in cancers of lymphatic lineage. Patient-derived xenograft (pdx) mouse models of these malignancies are available for investigation. As noted above, glucocorticoids can induce apoptosis in these malignancies, but their application is limited by their toxic effects, which are not shared by beta2-adrenergic related drugs, as already discussed [67,68,69]. We have already reported that phenoxybenzamine can inhibit proliferation of cultures of two cell types of lymphatic lineage, lymphoma cells SU-DHL-1 and myeloma RPMI 8226 [43].

## 5. Conclusions

Advances in the development of beta2-selective adrenergic agonists has led to their recognition as potent anti-inflammatory agents with potential for treatment in Parkinson’s disease and inflammatory disorders. This potential was reinforced in the present study by observations that epinephrine, the prototype beta2-adrenergic agonist, showed an almost identical gene-expression signature to that of cortisol (hydrocortisone) and other glucocorticoids.

Levalbuterol and other beta2-adrenergic-related agents inhibited the release of eotaxin, VEGFa, and IL-13 from mice engrafted with human peripheral blood monocyte cultures in a Jackson Laboratory assay, supporting predictions of similar gene-expression mechanisms and potential translation to inflammatory pathologies and neoplastic syndromes. The observations with the group of drugs that were studied suggested a unifying mechanism of action of maintenance or elevation in cellular levels of cAMP. This aspect requires further investigation.

While therapeutic efforts in neurodegenerative, inflammatory, and neoplastic diseases that block downstream pathways and receptors have advanced considerably, there remain the issues of development of tolerance, escape mechanisms, and off-target adverse effects. There may be a philosophical advantage to inhibition of the release of inciting elements in these pathologies, as suggested by the present studies.

The agents that were studied are FDA-approved drugs with minimal adverse effect profiles. The fact that they showed no toxicity in the animal studies at doses translated from human experience, and that there are existing formulations that can be administered orally, may facilitate their repurposing and ultimate translation to appropriate pathologies.

## Figures and Tables

**Table 1:** Cell Lines Profiled in the Touchstone Database.

Cell Line	Description
A375	Human malignant melanoma
A549	Human non-small cell carcinoma
HA1E	Human kidney epithelial immortalized
HCC515	Human non-small cell lung adenocarcinoma
HEPG2	Human hepatocellular carcinoma cell line
MCF7	Human breast adenocarcinoma
PC3	Human prostate adenocarcinoma
VCAP	Human metastatic prostate cancer
HT29	Human colorectal adenocarcinoma

**Table 2:** Experimental Protocol, Dosages, and Interventions.

Group	N	Drinking Water * (D4)	Compound (D6)	Dose (mg/kg)	Dosing Route	Dosing Frequency	Bleed **	
							RO	Terminal
1	5	No pre-treatment	PBS	N/A	IV	Single dose	D6	D9
2	5		OKT3	0.25	IV	Single dose	D6	D9
3	5		Anti-CD28	1	IV	Single dose	D6	D9
4	5	Albuterol 8 ug/mL	PBS	N/A	IV	Single dose	D6	D9
5	5		OKT3	0.25	IV	Single dose	D6	D9
6	5		Anti-CD28	1	IV	Single dose	D6	D9
7	5	Levalbuterol 4 ug/mL	PBS	N/A	IV	Single dose	D6	D9
8	5		OKT3	0.25	IV	Single dose	D6	D9
9	5		Anti-CD28	1	IV	Single dose	D6	D9
10	5	Phenoxy-benzamine 5 ug/mL	PBS	N/A	IV	Single dose	D6	D9
11	5		OKT3	0.25	IV	Single dose	D6	D9
12	5		Anti-CD28	1	IV	Single dose	D6	D9
13	5	Theophylline 400 ug/mL	PBS	N/A	IV	Single dose	D6	D9
14	5		OKT3	0.25	IV	Single dose	D6	D9
15	5		Anti-CD28	1	IV	Single dose	D6	D9
16	5	Arformoterol tartrate 0.008 ug/mL	PBS	N/A	IV	Single dose	D6	D9
17	5		OKT3	0.25	IV	Single dose	D6	D9
18	5		Anti-CD28	1	IV	Single dose	D6	D9
Total	90							

* Freshly prepared drinking water was provided daily. ** Mice were bled via retro-orbital (RO) on Day 6 and cardiac puncture on Day 9. N/A: No dose is applicable; this is the phosphate buffered saline medium, the control treatment.

**Table 3:** CMap Class Connectivity: Epinephrine.

Perturbagen Class (PCL)	Group Enrichment Score
Progesterone receptor agonist × 12	100.00
Glucocorticoid receptor agonist × 44	99.67
Aromatase inhibitor × 3	97.00
Beta-adrenergic receptor agonist × 11	96.12
FLT3 inhibitor × 6	95.46
Progesterone receptor antagonist × 5	95.13
Leucine rich repeat kinase inhibitor × 3	94.15
Bromodomain inhibitor × 6	93.59
DNA synthesis inhibitor × 3	92.13
VEGFR inhibitor × 13	91.84
RAF inhibitor × 9	91.19

Sets of compound perturbagens with enrichment scores above 90 (similar) and below −90 (opposing) in relation to their correspondence with the gene-expression signatures of epinephrine.

**Table 4:** CMap Class Connectivity: Norepinephrine.

Perturbagen Class (PCL)	Group Enrichment Score
Beta-adrenergic receptor agonist × 11	99.95
Adenosine receptor agonist × 6	99.58
Reverse transcriptase inhibitor × 4	99.27
Bacterial 30S ribosomal subunit inhibitor × 5	99.26
GABA receptor antagonist × 5	95.90
Sigma receptor antagonist × 3	95.58
Bile acid × 4	93.38
PPAR receptor agonist × 16	92.04
Benzodiazepine receptor agonist × 4	90.55
FXR antagonist × 3	90.27
Estrogen receptor agonist × 17	90.13

Sets of compound perturbagens with enrichment scores above 90 (similar) and below −90 (opposing) in relation to their correspondence with the gene-expression signatures of norepinephrine.

**Table 5:** Gene-Expression Connectivity Scores for Epinephrine vs. GR Agonists.

Rank	Score	Name	Description
4	99.89	Hydrocortisone	Glucocorticoid receptor agonist
5	99.89	Betamethasone	Glucocorticoid receptor agonist
7	99.79	Budesonide	Glucocorticoid receptor agonist
8	99.79	Flunisolide	Cytochrome P450 inhibitor
9	99.75	Mometasone	Glucocorticoid receptor agonist
11	99.75	Fluocinonide	Glucocorticoid receptor agonist
13	99.68	Triamcinolone	Glucocorticoid receptor agonist
14	99.68	Fludroxycortide	Glucocorticoid receptor agonist
19	99.62	Clobetasol	Glucocorticoid receptor agonist
21	99.61	Triamcinolone	Glucocorticoid receptor agonist
22	99.61	Flumetasone	Glucocorticoid receptor agonist
23	99.58	Fluticasone	Glucocorticoid receptor agonist
24	99.58	Fluorometholone	Glucocorticoid receptor agonist
26	99.54	Dexamethasone	Glucocorticoid receptor agonist
27	99.52	Prednicarbate	Phospholipase activator
28	99.51	Isoflupredone	Glucocorticoid receptor agonist
29	99.47	Hydrocortisone	Glucocorticoid receptor agonist
30	99.44	Diflorasone	Corticosteroid agonist
31	99.44	Dexamethasone	Glucocorticoid receptor agonist
32	99.4	Medrysone	Glucocorticoid receptor agonist
33	99.37	Prednisolone	Glucocorticoid receptor agonist
34	99.33	Halcinonide	Glucocorticoid receptor agonist
37	99.12	Betamethasone	Glucocorticoid receptor agonist
39	99.05	Beclomethasone	Glucocorticoid receptor agonist
40	99.05	Fluocinolone	Glucocorticoid receptor agonist
42	99.05	Hydrocortisone	Glucocorticoid receptor agonist
43	99.01	Hydrocortisone	Glucocorticoid receptor agonist
45	98.77	Clocortolone	Glucocorticoid receptor agonist
46	98.77	Beclomethasone	Glucocorticoid receptor agonist
48	98.6	Westcort	Glucocorticoid receptor agonist
51	98.4	Prednisolone	Glucocorticoid receptor agonist
52	98.38	Fluticasone	Glucocorticoid receptor agonist
55	98.34	Hydrocortisone	Glucocorticoid receptor agonist
56	98.32	Rimexolone	Glucocorticoid receptor agonist
58	98.17	Fludrocortisone	Glucocorticoid receptor agonist
59	98.17	Amcinonide	Glucocorticoid receptor agonist
61	98.03	Methylprednisolone	Glucocorticoid receptor agonist
67	97.71	Desoximetasone	Glucocorticoid receptor agonist
75	97.67	Halometasone	Glucocorticoid receptor agonist
88	97.53	Loteprednol	Glucocorticoid receptor agonist
96	97.5	Depomedrol	Glucocorticoid receptor agonist
117	97.39	Prednisolone	Glucocorticoid receptor agonist
137	97.15	Fluocinonide	Glucocorticoid receptor agonist
632	93.24	Alclometasone	Glucocorticoid receptor agonist

**Table 6:** Gene-Expression Connectivity Scores for Norepinephrine vs. GR agonists.

Rank	Score	Name	Description
23	99.55	Fluocinonide	Glucocorticoid receptor agonist
244	97.47	Depomedrol	Glucocorticoid receptor agonist
376	96.44	Beclometasone	Glucocorticoid receptor agonist
471	95.46	Fluocinolone	Glucocorticoid receptor agonist
615	94.13	Prednisolone	Glucocorticoid receptor agonist
890	91.89	Triamcinolone	Glucocorticoid receptor agonist
943	91.4	Amcinonide	Glucocorticoid receptor agonist
1479	86.61	Rimexolone	Glucocorticoid receptor agonist
1810	83.17	Hydrocortisone	Glucocorticoid receptor agonist
1959	82.28	Halometasone	Glucocorticoid receptor agonist
2159	80.37	Beclometasone	Glucocorticoid receptor agonist
2349	78.38	Triamcinolone	Glucocorticoid receptor agonist
2445	77.7	Prednisolone	Glucocorticoid receptor agonist
2855	73.57	Betamethasone	Glucocorticoid receptor agonist
2864	73.36	Hydrocortisone	Glucocorticoid receptor agonist
2877	73.18	Alclometasone	Glucocorticoid receptor agonist
3084	71.41	Westcort	Glucocorticoid receptor agonist
3155	70.7	Fluticasone	Glucocorticoid receptor agonist
3160	70.64	Loteprednol	Glucocorticoid receptor agonist
3348	68.45	Dexamethasone	Glucocorticoid receptor agonist
3561	66.22	Fluocinonide	Glucocorticoid receptor agonist
3591	65.9	Desoximetasone	Glucocorticoid receptor agonist
4051	61.03	Betamethasone	Glucocorticoid receptor agonist
4147	60	Prednicarbate	Phospholipase activator
4393	56.96	Clocortolone	Glucocorticoid receptor agonist
4404	56.86	Hydrocortisone	Glucocorticoid receptor agonist
4465	56.37	Fluorometholone	Glucocorticoid receptor agonist
4489	56.05	Flunisolide	Cytochrome P450 inhibitor
4701	52.82	Mometasone	Glucocorticoid receptor agonist
5218	45.35	Dexamethasone	Glucocorticoid receptor agonist
5512	41.08	Prednisolone	Glucocorticoid receptor agonist
5594	39.97	Fludroxycortide	Glucocorticoid receptor agonist
5618	39.72	Methylprednisolone	Glucocorticoid receptor agonist
5696	38.68	Fluticasone	Glucocorticoid receptor agonist
5837	36.6	Hydrocortisone	Glucocorticoid receptor agonist
6223	31.46	Medrysone	Glucocorticoid receptor agonist
6329	29.69	Budesonide	Glucocorticoid receptor agonist
6560	25.98	Isoflupredone	Glucocorticoid receptor agonist
6962	19.87	Clobetasol	Glucocorticoid receptor agonist
7001	19.45	Fludrocortisone	Glucocorticoid receptor agonist
7129	16.81	Halcinonide	Glucocorticoid receptor agonist
8126	−10.56	Flumetasone	Glucocorticoid receptor agonist
8351	−40.7	Hydrocortisone	Glucocorticoid receptor agonist
8433	−53.42	Diflorasone	Corticosteroid agonist

**Table 7:** Characteristics of Subtypes of Adrenergic Receptors ^1^. Modified from Goodman and Gilman’s *The Pharmacological Basis of Therapeutics*, 9th Edition, 1996. Joel G. Hardman and Lee E. Limbird, Eds. McGraw-Hill, New York. Chapter 6, Neurotransmission, Page 125 (Table 6-3).

Receptor	Agonists	Antagonists	Tissue	Responses
Alpha1	Epi ≥ NE >> Iso Drug example, phenylephrine	Prazosin	Vascular smooth muscle	Contraction
			Genitourinary smooth muscle	Contraction
			Liver	Glycogenolysis; gluconeogenesis
			Intestinal smooth muscle	Hyperpolarization and relaxation
			Heart	Increased contractile force, arrhythmias
Alpha2	Epi ≥ NE >> Iso Drug example, Clonidine	Yohimbine	Pancreatic islet (b cells)	Decreased insulin secretion
			Platelets	Aggregation
			Nerve terminals	Decreased release of NE
			Vascular Smooth Muscle	Contraction
Beta1	Iso >> Epi = NE Drug example, Dobutamine	Metoprolol	Heart Juxtaglomerular cell	Increased force and rate of contraction and AV nodal conduction velocity Increased renin secretion
Beta2	Iso >> Epi >>> NE Drug example, Terbutaline	ICI 118551 (Inverse agonist)	Smooth muscle (vascular, bronchial, gastrointestinal, and genitourinary)	Relaxation
			Skeletal muscle	Glycogenolysis; uptake of K+
			Liver	Glycogenolysis; gluconeogenesis
Beta3	Iso = NE > Epi	ICI 118551	Adipose tissue	Lipolysis

^1^ This table provides examples of drugs that act on adrenergic receptors and of the locations of subtypes of adrenergic receptors. Abbreviations: epinephrine (Epi); norepinephrine (NE); isoproterenol (Iso). Epi ≥ NE >> Iso (Epi equal to/slightly greater than NE, which is greater than Iso); Iso >> Epi = NE (Iso greater than Epi, which is equal to NE); Iso >> Epi >>> NE (Iso greater than Epi, which is much greater than NE); Iso = NE > Epi (Iso equal to NE, which is slightly greater than Epi).

**Table 8:** CMap Class Connectivity: Hydrocortisone.

Perturbagen Class (PCL)	Group Enrichment Score
Glucocorticoid receptor agonist × 44	99.89
Cyclooxygenase inhibitor × 6	97.34
Beta-adrenergic receptor agonist × 11	95.88
Adenosine receptor agonist × 6	95.49
Na-K-Cl transporter inhibitor × 3	94.89
Rho associated kinase inhibitor × 4	93.79
MEK inhibitor × 8	92.61

Sets of compound perturbagens with enrichment scores above 90 (similar) and below −90 (opposing) in relation to their correspondence with the gene-expression signatures of hydrocortisone.

**Table 9:** Gene-expression connectivity scores for Hydrocortisone vs. GR agonists.

Rank	Score	Name	Description
1	99.99	Hydrocortisone	Glucocorticoid receptor agonist
2	99.93	Triamcinolone	Glucocorticoid receptor agonist
3	99.93	Budesonide	Glucocorticoid receptor agonist
4	99.93	Fluocinonide	Glucocorticoid receptor agonist
5	99.93	Fluorometholone	Glucocorticoid receptor agonist
7	99.72	Mometasone	Glucocorticoid receptor agonist
8	99.72	Fluticasone	Glucocorticoid receptor agonist
9	99.72	Fludrocortisone	Glucocorticoid receptor agonist
11	99.61	Betamethasone	Glucocorticoid receptor agonist
12	99.61	Betamethasone	Glucocorticoid receptor agonist
13	99.58	Clocortolone	Glucocorticoid receptor agonist
14	99.58	Flunisolide	Cytochrome P450 inhibitor
15	99.58	Dexamethasone	Glucocorticoid receptor agonist
16	99.58	Clobetasol	Glucocorticoid receptor agonist
17	99.54	Isoflupredone	Glucocorticoid receptor agonist
20	99.37	Fludroxycortide	Glucocorticoid receptor agonist
21	99.33	Prednisolone	Glucocorticoid receptor agonist
22	99.3	Beclomethasone	Glucocorticoid receptor agonist
23	99.3	Fluticasone	Glucocorticoid receptor agonist
24	99.3	Alclometasone	Glucocorticoid receptor agonist
26	99.3	Hydrocortisone	Glucocorticoid receptor agonist
27	99.26	Fluocinolone	Glucocorticoid receptor agonist
28	99.25	Flumetasone	Glucocorticoid receptor agonist
30	99.16	Rimexolone	Glucocorticoid receptor agonist
32	99.06	Loteprednol	Glucocorticoid receptor agonist
34	98.91	Hydrocortisone	Glucocorticoid receptor agonist
35	98.8	Halcinonide	Glucocorticoid receptor agonist
36	98.66	Triamcinolone	Glucocorticoid receptor agonist
37	98.63	Dexamethasone	Glucocorticoid receptor agonist
38	98.59	Beclomethasone	Glucocorticoid receptor agonist
39	98.52	Prednicarbate	Phospholipase activator
44	98.48	Prednisolone	Glucocorticoid receptor agonist
45	98.48	Amcinonide	Glucocorticoid receptor agonist
46	98.1	Halometasone	Glucocorticoid receptor agonist
48	97.82	Methylprednisolone	Glucocorticoid receptor agonist
50	97.68	Diflorasone	Corticosteroid agonist
52	97.5	Hydrocortisone	Glucocorticoid receptor agonist
53	97.5	Fluocinonide	Glucocorticoid receptor agonist
55	97.36	Desoximetasone	Glucocorticoid receptor agonist
58	97.33	Prednisolone	Glucocorticoid receptor agonist
66	97.15	Depomedrol	Glucocorticoid receptor agonist
83	96.69	Medrysone	Glucocorticoid receptor agonist
141	95.45	Westcort	Glucocorticoid receptor agonist
187	95.1	Hydrocortisone	Glucocorticoid receptor agonist

**Table 10:** CMap Class Connectivity: Phenoxybenzamine.

Perturbagen Class (PCL)	Group Enrichment Score
Progesterone receptor agonist × 12	99.50
Glucocorticoid receptor agonist × 44	98.78
Aromatase inhibitor × 3	97.98
Glycogen synthase kinase inhibitor × 11	97.63
HSP inhibitor × 7	96.76
EGFR inhibitor × 14	96.33
PPAR receptor agonist × 16	95.40
DNA synthesis inhibitor × 3	95.02
PKC inhibitor × 7	93.75
JAK inhibitor × 5	92.59
HIV protease inhibitor × 4	92.34
Cannabinoid receptor agonist × 8	91.13

Sets of compound perturbagens with enrichment scores above 90 (similar) and below −90 (opposing) in relation to their correspondence with the gene-expression signatures of phenoxybenzamine.

**Table 11:** CMap Class Connectivity: Theophylline.

Perturbagen Class (PCL)	Group Enrichment Score
Dihydrofolate reductase inhibitor × 4	99.78
Reverse transcriptase inhibitor × 4	99.40
Glucocorticoid receptor agonist × 44	98.37
Vitamin D receptor agonist × 6	97.36
Nucleoside reverse transcriptase inhibitor × 3	97.07
IMPDH inhibitor × 3	95.65
Bacterial 30S ribosomal subunit inhibitor × 5	94.42
FXR antagonist × 3	93.95
Calmodulin antagonist × 3	93.51
Bacterial cell wall synthesis inhibitor × 9	90.77

Sets of compound perturbagens with enrichment scores above 90 (similar) and below −90 (opposing) in relation to their correspondence with the gene-expression signatures of theophylline.

**Table 12:** CMap Class Connectivity: Caffeine.

Perturbagen Class (PCL)	Group Enrichment Score
Bacterial DNA gyrase inhibitor × 5	95.54
Aromatase inhibitor × 3	93.32
Dihydrofolate reductase inhibitor × 4	91.76
Cyclooxygenase inhibitor × 6	90.65
Glucocorticoid receptor agonist × 44	90.24

Sets of compound perturbagens with enrichment scores above 90 (similar) and below −90 (opposing) in relation to their correspondence with the gene-expression signatures of caffeine.

## Data Availability

All the essential data are included in the manuscript. Body weights for individual animals and water consumption data by cage are available upon request.

## Supplementary Materials

The following supporting information can be downloaded at https://www.mdpi.com/article/10.3390/biomedicines12081720/s1, Table S1: Gene-expression connectivity scores of adrenergic receptor agonists in relation to salbutamol. Table S2: Gene-expression connectivity scores of dopamine receptor agonists in relation to salbutamol. Table S3: Gene-expression connectivity scores for Phenoxybenzamine vs. GR agonists. Table S4. Glucocorticoid receptor agonist list with targets. Table S5. Gene-expression connectivity scores for Theophylline vs. GR agonists. Table S6. Gene-expression connectivity scores for Caffeine vs. GR agonists. Figure S1. Association between rank score and gene-expression connectivity score.
