# Supplementary material for: Beta2-Adrenergic Suppression of Neuroinflammation in Treatment of Parkinsonism, with Relevance for Neurodegenerative and Neoplastic Disorders
Source: Biomedicines. 2024 Aug 1;12(8):1720. doi: 10.3390/biomedicines12081720 (PMC11351568; doi:10.3390/biomedicines12081720)
Supplement: Supplementary file 1 [file biomedicines-12-01720-s001.zip › Figure S1 EDITED.pdf]

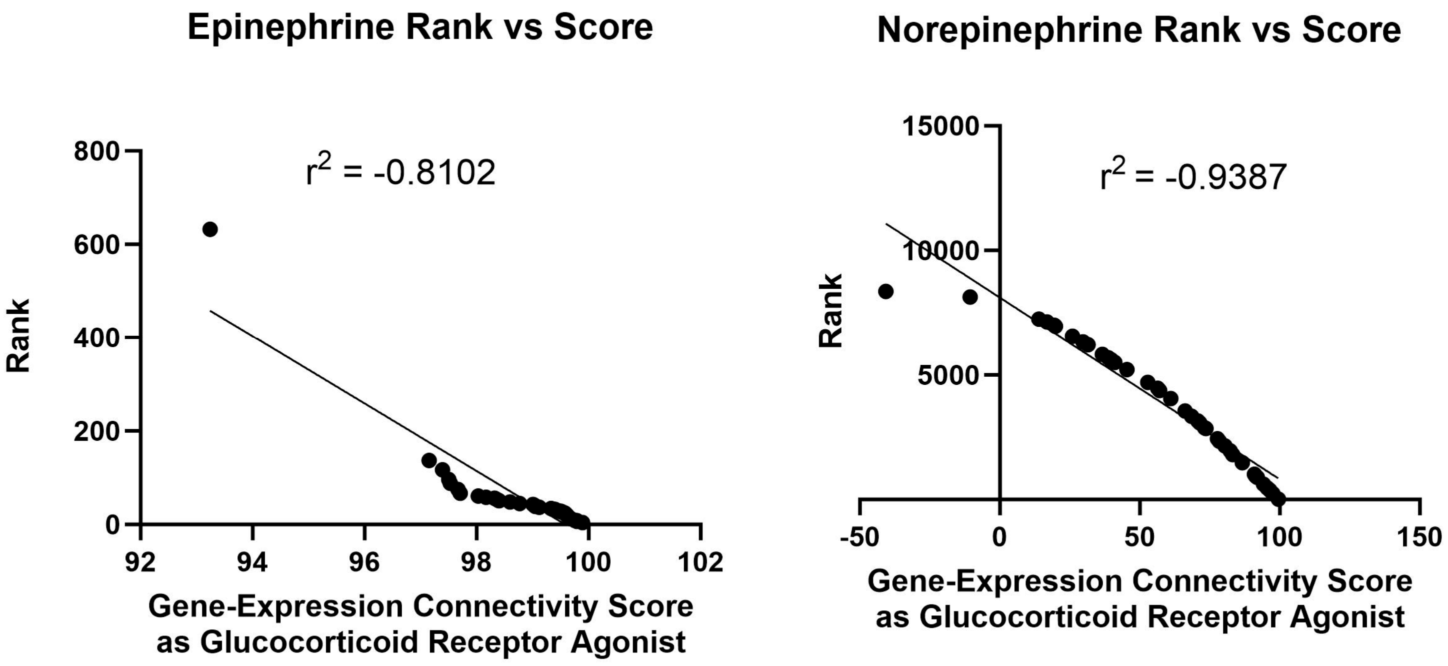

Figure S1. Comparison of the associations between the Rank and the Gene-Expression Connectivity Scores for glucocorticoid receptor agonist activity for epinephrine and norepinephrine in the CLUE database. Both associations show highly significant inverse relationships for the two measures. However, the beta2-adrenergic prototype, epinephrine, shows a much closer relationship between rank and the gene-expression connectivity scores. Norepinephrine lacks significant beta-2 adrenergic activity (Table 7). The data for these two measures for epinephrine and norepinephrine are presented in Tables 5 and 6, respectively. A plot demonstrating these differences is presented in Figure 3. In practical terms, "rank" represents the relative depth that the CLUE database must be probed to find gene-expression connectivity with perturbagens; the further the probe, the weaker the connectivity.
