# Supplementary material for: Beta2-Adrenergic Suppression of Neuroinflammation in Treatment of Parkinsonism, with Relevance for Neurodegenerative and Neoplastic Disorders
Source: Biomedicines. 2024 Aug 1;12(8):1720. doi: 10.3390/biomedicines12081720 (PMC11351568; doi:10.3390/biomedicines12081720)
Supplement: Supplementary file 1 [file biomedicines-12-01720-s001.zip › Table S1.pdf]

Table S1. Gene-expression connectivity scores of adrenergic receptor agonists in relation to salbutamol.

Rank	Score	Name	Description	Target
3	99.93	cimaterol	Adrenergic receptor agonist	ADRB3, ADRB1, ADRB2
27	99.37	procaterol	Adrenergic receptor agonist	ADRB2
118	97.54	dobutamine	Adrenergic receptor agonist	ADRB1, ADRB2, ADRA1A 3
183	96.72	orciprenaline	Adrenergic receptor agonist	ADRB2
255	95.52	buphenine	Adrenergic receptor agonist	ADRB2
288	95.21	fenoterol	Adrenergic receptor agonist	ADRB2, ADRB1, ADRB3
515	92.56	isoxsuprine	Adrenergic receptor agonist	ADRB2
553	92.25	salmeterol	Adrenergic receptor agonist	ADRB2

ADRB2, beta2-adrenergic receptor; ADRB1, beta1-adrenergic receptor; ADRA1A, alpha1a-adrenergic receptor; ADRB3, beta3-adrenergic receptor.
