# Supplementary material for: Beta2-Adrenergic Suppression of Neuroinflammation in Treatment of Parkinsonism, with Relevance for Neurodegenerative and Neoplastic Disorders
Source: Biomedicines. 2024 Aug 1;12(8):1720. doi: 10.3390/biomedicines12081720 (PMC11351568; doi:10.3390/biomedicines12081720)
Supplement: Supplementary file 1 [file biomedicines-12-01720-s001.zip › Table S2.pdf]

Table S2. Gene-expression connectivity scores of dopamine receptor agonists in relation to salbutamol.

Rank	Score	Name	Description	Target
8	99.65	SKF-77434	Dopamine receptor agonist	DRD1
51	98.70	fenoldopam	Dopamine receptor agonist	DRD1, ADRA1A, ADRA1B, ADRA1D, ADRA2A, ADRA2B, ADRA2C, DRD4, DRD5
52	98.66	pramipexole	Dopamine receptor agonist	DRD3, DRD2, ADRA2A, ADRA2B, ADRA2C, DRD1, DRD4, DRD5, HTR1A, HTR1B, HTR1D, HTR2A, HTR2B, HTR2C
335	93.45	RO-10-5824	Dopamine receptor agonist	DRD4
385	92.63	dopamine	Dopamine receptor agonist	DRD2, DRD1, DRD3, DRD4, DRD5, DBH, TR1A, HTR7, SLC6A2, SLC6A3, SLC6A4

DRDI, dopamine receptor D1; DRD2, dopamine receptor D2; DRD3, dopamine receptor D3; DRD4, dopamine receptor D4; DRD5, dopamine receptor D5; ADRA1A, alpha1a-adrenergic receptor; ADRA1B, alpha1b-adrenergic receptor; ADRA1D, alpha1d-adrenergic receptor; ADRA2A, alpha2a-adrenergic receptor; ADRA2B, alpha2b-adrenergic receptor; ADRA2C, alpha2c-adrenergic receptor; HTR1A, HTR1B, HTR1D, HTR2A, HTR2B, HTR2C, serotonin receptors 1A, 1B, 1D, 2A, 2B, 2C; DBH, dopamine beta-hydroxylase; TR1A, T regulatory Type 1 cells; HTR7, serotonin 7 receptor; SLC6A2, norepinephrine transporter; SLC6A3, sodium dependent dopamine transporter; SLC6A4. serotonin transporter.
