# Supplementary material for: Beta2-Adrenergic Suppression of Neuroinflammation in Treatment of Parkinsonism, with Relevance for Neurodegenerative and Neoplastic Disorders
Source: Biomedicines. 2024 Aug 1;12(8):1720. doi: 10.3390/biomedicines12081720 (PMC11351568; doi:10.3390/biomedicines12081720)
Supplement: Supplementary file 1 [file biomedicines-12-01720-s001.zip › Table S3.pdf]

Table S3. Gene-Expression Connectivity Scores for PHENOXYBENZAMINE vs GR agonists.

Rank	Score	Name	Description
**4**	99.93	Depomedrol	Glucocorticoid receptor agonist
**6**	99.93	Halcinonide	Glucocorticoid receptor agonist
**8**	99.93	Clocortolone	Glucocorticoid receptor agonist
**11**	99.93	Budesonide	Glucocorticoid receptor agonist
**12**	99.93	Prednisolone	Glucocorticoid receptor agonist
**13**	99.93	Fludroxycortide	Glucocorticoid receptor agonist
**15**	99.93	Fludrocortisone	Glucocorticoid receptor agonist
**18**	99.93	Amcinonide	Glucocorticoid receptor agonist
**20**	99.89	Beclomethasone	Glucocorticoid receptor agonist
**23**	99.82	Fluocinonide	Glucocorticoid receptor agonist
**33**	99.72	Mometasone	Glucocorticoid receptor agonist
**34**	99.72	Triamcinolone	Glucocorticoid receptor agonist
**46**	99.51	Beclomethasone	Glucocorticoid receptor agonist
**55**	99.44	Prednisolone	Glucocorticoid receptor agonist
**113**	98.27	Fluticasone	Glucocorticoid receptor agonist
**114**	98.27	Isoflupredone	Glucocorticoid receptor agonist
**127**	98.13	Westcort	Glucocorticoid receptor agonist
**141**	97.74	Hydrocortisone	Glucocorticoid receptor agonist
**163**	97.53	flunisolide	Cytochrome P450 inhibitor
**167**	97.5	Flumetasone	Glucocorticoid receptor agonist
**180**	97.38	Clobetasol	Glucocorticoid receptor agonist
**221**	96.45	Rimexolone	Glucocorticoid receptor agonist
**291**	95.26	Loteprednol	Glucocorticoid receptor agonist
**347**	93.94	Fluocinolone	Glucocorticoid receptor agonist
**598**	89.54	Prednisolone	Glucocorticoid receptor agonist
**769**	87.05	Alclometasone	Glucocorticoid receptor agonist
**779**	86.97	Diflorasone	Corticosteroid agonist
**814**	86.66	Betamethasone	Glucocorticoid receptor agonist
**882**	85.73	Prednicarbate	Phospholipase activator
**953**	84.66	Hydrocortisone	Glucocorticoid receptor agonist
**1310**	79.4	Betamethasone	Glucocorticoid receptor agonist
**1637**	74.67	Hydrocortisone	Glucocorticoid receptor agonist
**1655**	74.17	Fluorometholone	Glucocorticoid receptor agonist
**1702**	73.53	Hydrocortisone	Glucocorticoid receptor agonist
**1719**	73.16	Triamcinolone	Glucocorticoid receptor agonist
**1836**	71.31	Desoximetasone	Glucocorticoid receptor agonist
**1930**	69.94	Dexamethasone	Glucocorticoid receptor agonist
**2543**	61.53	fluticasone	Glucocorticoid receptor agonist
**2665**	59.91	Halometasone	Glucocorticoid receptor agonist
**3383**	47.82	Dexamethasone	Glucocorticoid receptor agonist
**3419**	47.37	Fluocinonide	Glucocorticoid receptor agonist
**4447**	33.18	Hydrocortisone	Glucocorticoid receptor agonist
**4539**	31.79	methylprednisolone	Glucocorticoid receptor agonist
**4566**	31.68	Medrysone	Glucocorticoid receptor agonist
