# Supplementary material for: Beta2-Adrenergic Suppression of Neuroinflammation in Treatment of Parkinsonism, with Relevance for Neurodegenerative and Neoplastic Disorders
Source: Biomedicines. 2024 Aug 1;12(8):1720. doi: 10.3390/biomedicines12081720 (PMC11351568; doi:10.3390/biomedicines12081720)
Supplement: Supplementary file 1 [file biomedicines-12-01720-s001.zip › Table S4.pdf]

Table S4. Glucocorticoid Receptor (GR) Agonist Class List with Targets.

Broad ID	Name	Description	Target
A46186775	hydrocortisone	GR agonist	ANXA1, NOS2, NR3C1, NR3C2
A92439610	triamcinolone	GR agonist	NR3C1, CYP3A5, CYP3A7, SERPINA6
A82238138	budesonide	GR agonist	NR3CI, CYP3A5, CYP3A7
A15297126	fluocinonide	GR agonist	NR3C1, SERPINA6, SMO
A13133631	fluorometholone	GR agonist	NR3C1
K60640630	mometasone	GR agonist	NR3C1
K14791739	fluticasone	GR agonist	NR3C1, CYP3A5, CYP3A7, NR3C2, PGR, PLA2G4A
A38749782	fludrocortisone	GR agonist	NR3C2, AR, NR3C1
A92177080	betamethasone	GR agonist	NR3C1
A02180903	betamethasone	GR agonist	NR3C1
K38003476	clocortolone	GR agonist	NR3C1, PLA2G1B
A65449987	flunisolide	GR agonist	NR3C1
A35108200	dexamethasone	GR agonist	ANXA1, CYP3A4, CYP3A5, NOS2, NR0B1, NR3C1, NR3C2
A26095496	clobetasol	GR agonist	NR3C1, PLA2G1B
K29173907	isoflupredone	GR agonist	NR3C1
A49765801	fludroxycortide	GR agonist	NR3C1, SERPINA6
A78391468	prednisolone	GR agonist	NR3C1, NR3C2, SERPINA6
K97810537	beclometasone	GR agonist	NR3C1, CYP3A5, GPR97, SERPINA6
K62310379	fluticasone	GR agonist	NR3C1, CYP3A5, CYP3A7, NR3C2, PGR, PLA2G4A
A90131694	alclometasone	GR agonist	NR3C1, SERPINA6
A25143711	hydrocortisone	GR agonist	ANXA1, NOS2, NR3C1, NR3C2
K94353609	fluocinolone	GR agonist	NR3C1, SERPINA6
A01346607	flumetasone	GR agonist	NR3C1, PLA2G1B
K31627533	rimexolone	GR agonist	NR3C1, SERPINA6
K39983086	loteprednol	GR agonist	NR3C1
A07000685	hydrocortisone	GR agonist	ANXA1, NOS2, NR3C1, NR3C2
K81709173	halcinonide	GR agonist	NR3C1
K53790871	triamcinolone	GR agonist	NR3C1, CYP3A5, CYP3A7, SERPINA6
A93424738	dexamethasone	GR agonist	ANXA1, CYP3A4, CYP3A5, NOS2, NR0B1, NR3C1, NR3C2
A17448384	beclometasone	GR agonist	NR3C1, CYP3A5, GPR97, SERPINA6
K46137903	prednicarbate	GR agonist	NR3C1, PLA2G1B
A27887842	prednisolone	GR agonist	NR3C1, NR3C2, SERPINA6
A16478930	amcinonide	GR agonist	NR3C1, ANXA1
K33312228	halometasone	GR agonist	NR3C1
K35240538	methylprednisolone	GR agonist	NR3C1
K17674993	diflorasone	GR agonist	NR3C1, PLASG1B
K73978287	hydrocortisone	GR agonist	ANXA1, NOS2, NR3C1, NR3C2
K57886322	fluocinonide	GR agonist	NR3C1, SERPINA6, SMO
K30697463	desoximetasone	GR agonist	NR3C1, PLA2G1B
A01643550	prednisolone	GR agonist	NR3C1, NR3C2, SERPINA6
K94070024	depomedrol	GR agonist	NR3C1
A20126139	medrysone	GR agonist	NR3C1
A23290232	westcort	GR agonist	NR3C1
A65767837	hydrocortisone	GR agonist	ANXA1, NOS2, NR3C1, NR3C2

NR3CI, glucocorticoid receptor; NR3C2, mineralocorticoid receptor; ANXA1, annexin A1; NOS2, nitric oxide synthase 2; CYP3A5, cytochrome P450 3A5; CYP3A7, cytochrome P450 3A7; SERPINA6, transcortin; SMO, smoothened/frizzled class receptor; PGR, progesterone receptor; PLA2G4A, cytosolic

phospholipase A2; AR, androgen receptor; PLA2G1B, phospholipase A2/group 1B; CYP3A4, cytochrome P450 3A4; NROB1, nuclear receptor/subfamily 0/group B/member 1; GPR97, adhesion G protein-coupled receptor G3; PLASG1B, RNA-binding protein.
