# Supplementary material for: Beta2-Adrenergic Suppression of Neuroinflammation in Treatment of Parkinsonism, with Relevance for Neurodegenerative and Neoplastic Disorders
Source: Biomedicines. 2024 Aug 1;12(8):1720. doi: 10.3390/biomedicines12081720 (PMC11351568; doi:10.3390/biomedicines12081720)
Supplement: Supplementary file 1 [file biomedicines-12-01720-s001.zip › Table S5.pdf]

Table S5. Gene expression connectivity scores for THEOPHYLLINE vs GR agonists.

Rank	Score	Name	Description
21	99.3	amcinonide	Glucocorticoid receptor agonist
69	98.37	fluocinonide	Glucocorticoid receptor agonist
139	97.7	halometasone	Glucocorticoid receptor agonist
201	97.11	westcort	Glucocorticoid receptor agonist
263	96.51	hydrocortisone	Glucocorticoid receptor agonist
411	94.65	fluocinolone	Glucocorticoid receptor agonist
434	94.46	mometasone	Glucocorticoid receptor agonist
502	93.75	loteprednol	Glucocorticoid receptor agonist
520	93.51	beclometasone	Glucocorticoid receptor agonist
618	92.18	dexamethasone	Glucocorticoid receptor agonist
659	91.48	beclometasone	Glucocorticoid receptor agonist
668	91.47	fluocinonide	Glucocorticoid receptor agonist
690	91.3	medrysone	Glucocorticoid receptor agonist
713	91.11	prednisolone	Glucocorticoid receptor agonist
883	89.94	halcinonide	Glucocorticoid receptor agonist
888	89.9	depomedrol	Glucocorticoid receptor agonist
925	89.63	clocortolone	Glucocorticoid receptor agonist
1043	88.6	desoximetasone	Glucocorticoid receptor agonist
1096	87.94	diflorasone	Corticosteroid agonist
1178	87.08	dexamethasone	Glucocorticoid receptor agonist
1190	87	prednisolone	Glucocorticoid receptor agonist
1232	86.55	alclometasone	Glucocorticoid receptor agonist
1283	85.89	betamethasone	Glucocorticoid receptor agonist
1318	85.69	fluticasone	Glucocorticoid receptor agonist
1329	85.62	fluticasone	Glucocorticoid receptor agonist
1509	83.66	methylprednisolone	Glucocorticoid receptor agonist
1880	79.47	budesonide	Glucocorticoid receptor agonist
1882	79.46	triamcinolone	Glucocorticoid receptor agonist
2057	77.14	isoflupredone	Glucocorticoid receptor agonist
2452	72.37	prednisolone	Glucocorticoid receptor agonist
2489	71.54	prednicarbate	Phospholipase activator
2632	70.01	hydrocortisone	Glucocorticoid receptor agonist
2876	66.84	clobetasol	Glucocorticoid receptor agonist
3203	63.09	fluorometholone	Glucocorticoid receptor agonist
3403	60.6	rimexolone	Glucocorticoid receptor agonist
3577	58.56	hydrocortisone	Glucocorticoid receptor agonist
3605	57.97	betamethasone	Glucocorticoid receptor agonist
3936	53.51	hydrocortisone	Glucocorticoid receptor agonist
4678	43.88	fludrocortisone	Glucocorticoid receptor agonist
5044	40.07	hydrocortisone	Glucocorticoid receptor agonist
6024	26.95	flunisolide	Cytochrome P450 inhibitor
6221	24.87	flumetasone	Glucocorticoid receptor agonist
7523	9.69	triamcinolone	Glucocorticoid receptor agonist
7817	6.77	fludroxycortide	Glucocorticoid receptor agonist
