# Supplementary material for: Beta2-Adrenergic Suppression of Neuroinflammation in Treatment of Parkinsonism, with Relevance for Neurodegenerative and Neoplastic Disorders
Source: Biomedicines. 2024 Aug 1;12(8):1720. doi: 10.3390/biomedicines12081720 (PMC11351568; doi:10.3390/biomedicines12081720)
Supplement: Supplementary file 1 [file biomedicines-12-01720-s001.zip › Table S6.pdf]

TableS6. Gene expression connectivity scores for CAFFEINE vs GR agonists.

Rank	Score	Name	Description
213	98.34	alclometasone	Glucocorticoid receptor agonist
266	98.06	beclomethasone	Glucocorticoid receptor agonist
294	97.84	fluticasone	Glucocorticoid receptor agonist
340	97.74	rimexolone	Glucocorticoid receptor agonist
342	97.74	budesonide	Glucocorticoid receptor agonist
369	97.6	diflorasone	Corticosteroid agonist
546	96.38	desoximetasone	Glucocorticoid receptor agonist
605	95.96	prednisolone	Glucocorticoid receptor agonist
801	94.26	isoflupredone	Glucocorticoid receptor agonist
802	94.26	betamethasone	Glucocorticoid receptor agonist
984	92.8	triamcinolone	Glucocorticoid receptor agonist
1146	91.13	clocortolone	Glucocorticoid receptor agonist
1238	90.46	loteprednol	Glucocorticoid receptor agonist
1297	89.85	methylprednisolone	Glucocorticoid receptor agonist
1357	89.37	prednicarbate	Phospholipase activator
1525	87.34	dexamethasone	Glucocorticoid receptor agonist
1924	82.44	prednisolone	Glucocorticoid receptor agonist
1990	81.65	dexamethasone	Glucocorticoid receptor agonist
2060	80.88	beclomethasone	Glucocorticoid receptor agonist
2128	80.38	fluocinolone	Glucocorticoid receptor agonist
2164	79.94	flumetasone	Glucocorticoid receptor agonist
2431	77.42	hydrocortisone	Glucocorticoid receptor agonist
2515	76.32	clobetasol	Glucocorticoid receptor agonist
3163	67.41	halometasone	Glucocorticoid receptor agonist
3761	58.11	fludroxycortide	Glucocorticoid receptor agonist
3832	57.19	westcort	Glucocorticoid receptor agonist
3839	57.12	prednisolone	Glucocorticoid receptor agonist
3873	56.65	medrysone	Glucocorticoid receptor agonist
4311	50.09	depomedrol	Glucocorticoid receptor agonist
4632	45.21	mometasone	Glucocorticoid receptor agonist
4731	44.04	fluocinonide	Glucocorticoid receptor agonist
4870	42.07	hydrocortisone	Glucocorticoid receptor agonist
4954	40.88	hydrocortisone	Glucocorticoid receptor agonist
5046	39.65	hydrocortisone	Glucocorticoid receptor agonist
5467	34.15	amcinonide	Glucocorticoid receptor agonist
5826	28.63	fluorometholone	Glucocorticoid receptor agonist
5924	27.31	triamcinolone	Glucocorticoid receptor agonist
6059	24.86	halcinonide	Glucocorticoid receptor agonist
6203	22.66	betamethasone	Glucocorticoid receptor agonist
6372	19.84	flunisolide	Cytochrome P450 inhibitor
6673	15.86	hydrocortisone	Glucocorticoid receptor agonist
6678	15.81	fluticasone	Glucocorticoid receptor agonist
7332	5.38	fludrocortisone	Glucocorticoid receptor agonist
7492	3	fluocinonide	Glucocorticoid receptor agonist
